# A Content Analysis of Digital Marketing Strategies of Formula Companies and Influencers to Promote Commercial Milk Formula in Hong Kong

**DOI:** 10.1111/mcn.70007

**Published:** 2025-02-25

**Authors:** Wan Ching Ng, Karene Hoi Ting Yeung, Lai Ling Hui, Ka Ming Chow, Esther Yuet Ying Lau, E. Anthony S. Nelson

**Affiliations:** ^1^ Jockey Club School of Public Health and Primary Care, Faculty of Medicine The Chinese University of Hong Kong New Territories Hong Kong; ^2^ Department of Paediatrics, Faculty of Medicine The Chinese University of Hong Kong New Territories Hong Kong; ^3^ Department of Food Science and Nutrition The Hong Kong Polytechnic University Kowloon Hong Kong; ^4^ The Nethersole School of Nursing, Faculty of Medicine The Chinese University of Hong Kong New Territories Hong Kong; ^5^ Department of Psychology The Education University of Hong Kong New Territories Hong Kong; ^6^ School of Medicine Shenzhen Guangdong China

**Keywords:** breastfeeding, breast‐milk substitutes, commercial milk formula, content analysis, digital marketing, formula milk, Hong Kong, International Code of Marketing of Breast‐milk Substitutes

## Abstract

This study examines the compliance of the digital marketing strategies used by formula companies and social media influencers in promoting commercial milk formula (CMF) for infants and young children with the International Code of Marketing of Breast‐milk Substitutes (International Code) and the Hong Kong Code of Marketing of Formula Milk and Related Products, and Food Products for Infants & Young Children (HK Code). Content analyses on influencers' posts, websites and social media sites of five major formula companies were conducted to identify the digital marketing strategies used, evidence of product cross‐promotion, and compliance with the International and HK Codes. Statistical analyses were performed to assess the associations between marketing strategies and social media interactions (likes/comments/shares). The findings revealed widespread noncompliance to the two codes by both influencers and formula companies. Of 1036 influencer (*n* = 493) and formula company (*n* = 543) materials mentioned products covered by the Codes, all influencer materials and 95% of company materials violated at least two provisions of the Codes. Persistent brand promotion and cross‐promotion strategies were observed in CMF marketing in Hong Kong, with tactics such as parents' sharing, showing images of happy children and families, and celebrity endorsements leading to higher social media interactions. The extensive CMF promotion by influencers and the covert cross‐promotion and brand promotion are of particular concern given the lack of regulation in this area. The study calls for a comprehensive review and introduction of legalisation in Hong Kong to govern CMF marketing, ensuring enforcement as outlined in the Convention on the Rights of the Child.

## Summary

- Digital marketing of commercial milk formula products in Hong Kong violated both the International and Hong Kong Codes.
- Almost all materials by influencers and formula companies violated at least two provisions in the codes.
- The extensive promotion of commercial milk formula products by influencers is of particular concern given the lack of regulation in this area.
- Covert cross‐promotion and brand promotion of formula milk products was widespread in Hong Kong.
- A review and legalisation to govern the marketing of commercial milk formula is needed to protect breastfeeding.

## Graphical abstract

International and Hong Kong Codes that aim to control the marketing and promotion of commercial milk formula were extensively violated by both manufacturers and social media influencers in Hong Kong, highlighting the urgent need for enforceable legislation to protect breastfeeding.

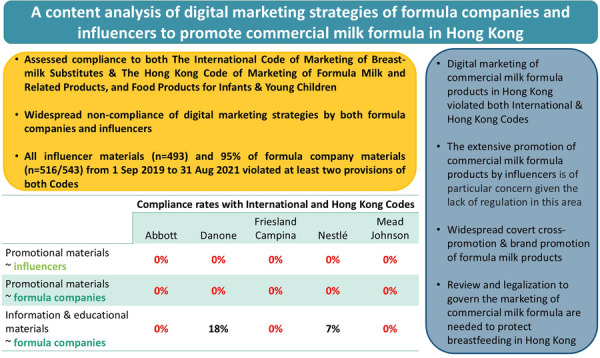

**Abbreviations**
BMS: breast‐milk substitutes
CMF: commercial milk formula
DH: Department of Health
HK Code: Hong Kong Code of Marketing of Formula Milk and Related Products, and Food Products for Infants & Young Children
IBFAN: International Baby Food Action Network
International Code: International Code of Marketing of Breast‐milk Substitutes
I&E: informational and educational
WHA: World Health Assembly
WHO: World Health Organization

## 1 Introduction

The World Health Organization (WHO) recommends mothers to exclusively breastfeed infants for the first 6 months of life, and continue breastfeeding with complementary feeding for up to 2 years and beyond (World Health Organization & United Nations Children's Fund 2003). The ever breastfeeding rate at hospital discharge in Hong Kong has been stable at 87% from 2016 to 2020 (Department of Health 2021a). However, the number of mothers who exclusively breastfed their babies to 6 months dropped from 28% to 22% during this period and those who sustained breastfeeding to 12 months decreased from 28% to 24%.

Breastfeeding is influenced by many factors, including commercial milk formula (CMF) marketing (e.g., advertisements of formula milk and provision of free infant formula), individual factors (e.g., mothers' attitudes and self‐efficacy towards breastfeeding, and maternal knowledge about breastfeeding), family (e.g., mental and physical support from husband and other family members and their beliefs towards breastfeeding), health systems and health workers (e.g., accessibility to maternal health care and breastfeeding counselling, mother‐baby‐friendly hospital policies and practices, health care workers' knowledge and supportiveness towards breastfeeding), and societal factors (e.g., social norms, maternal leave policies and fiscal policies) (Asimaki et al. 2022; Alive et al. 2022; Baker et al. 2023; Nasrabadi et al. 2019; Piwoz and Huffman 2015). Breast‐milk substitutes (BMS) are any food that is represented or promoted as a total or partial replacement of breast‐milk (World Health Organization 1981), and its promotion influences social norms and people's attitudes towards breastfeeding. By promoting the health and developmental benefits of CMF (e.g., highlighting babies will be smart and grow better after consuming the CMF products) and its comparability to breastmilk (Piwoz and Huffman 2015) (e.g., highlighting that the CMF products contain ingredients that mimic breast‐milk), the duration and exclusivity of breastfeeding have been undermined (Kaplan and Graff 2008; Piwoz and Huffman 2015). Furthermore, the CMF marketing often exploits mothers' insecurities about their milk and their ability to satiate and to calm their babies, and promote CMF as a solution to unsettled babies. This would encourage parents to introduce CMF early and thus reduce the likelihood of successful breastfeeding (Pérez‐Escamilla et al. 2023), eventually leading to suboptimal breastfeeding practices.

Suboptimal breastfeeding contributes to multiple health impacts to infants, young children and mothers, including infant malnutrition, increased risks of child diarrhoea, acute respiratory illness and obesity, increased risk of developing chronic diseases later in the children's life, increased mortality among infants and young children, and increased risks for mothers to develop chronic diseases such as cardiovascular diseases, type 2 diabetes, and breast and ovarian cancers due to loss of protection from breastfeeding (Pérez‐Escamilla et al. 2023; Alive and Thrive 2022). Nonetheless, suboptimal breastfeeding can also lead to some socioeconomic impacts such as loss of human capital, and increased costs and burdens to the health system due to mortality and morbidity from the unrealised health and developmental benefits of breastfeeding (Pérez‐Escamilla et al. 2023; Alive and Thrive 2022). In addition, CMF marketing hinders access to truthful and impartial information, which is an essential human right stated in the United Nations Convention on the Rights of the Child (The Office of the United Nations High Commissioner for Human Rights 1989; Rollins et al. 2023).

To protect breastfeeding from inappropriate BMS marketing, WHO adopted the International Code of Marketing of Breast‐milk Substitutes (International Code) in 1981 (World Health Organization 1981) and several World Health Assembly (WHA) resolutions subsequent to the International Code to clarify and update certain provisions of the original International Code (World Health Organization 2024). As of 2022, around 75% of the WHO Member States have adopted at least some provisions of the International Code and the subsequent WHA resolutions into their legal measures (World Health Organization 2022a). In 2017, a voluntary, non‐legally binding Hong Kong Code of Marketing of Formula Milk and Related Products, and Food Products for Infants & Young Children (HK Code) was enacted by the Hong Kong Government (Health Bureau, Department of Health 2017). The two codes apply to the marketing practices related to formula milk, foods and beverages that are represented or promoted as BMS for children aged below 36 months and cover several aspects, including information and education on infant feeding and promotion to the public and mothers, and so forth (Health Bureau, Department of Health 2017; World Health Organization 1981, 2024).

Many overseas studies have identified inappropriate CMF marketing practices that violated the International Code (Abrahams 2012; Baker et al. 2023; Becker et al. 2022; Chen et al. 2015; Ching et al. 2021; Pereira‐Kotze et al. 2020; Pérez‐Escamilla et al. 2023; Rollins et al. 2023; Vinje et al. 2017; World Health Organization 2022b, 2022c), and six local studies have assessed CMF marketing in Hong Kong (Department of Health 2013, 2016; 2017, 2021b; Globalization Monitor 2019; Mak 2015). These studies identified some common marketing strategies by formula companies including: (i) use of rational appeals such as health or nutrition claims (Chen et al. 2015; Ching et al. 2021; Department of Health 2017; Globalization Monitor 2019), promotional offers (Ching 2017; Department of Health 2017, 2021b), scientific or medical evidence, and professional endorsement (Chen et al. 2015; Department of Health 2017, 2021b); (ii) use of emotional appeals like celebrity endorsement (Chen et al. 2015; Department of Health 2017, 2021b), parent‐child bonding, and happy child (Chen et al. 2015; Department of Health 2017, 2021b; Globalization Monitor 2019); and (iii) cross‐promotion (Ching 2017; Department of Health 2017, 2021b; Globalization Monitor 2019), which is the indirect promotion of CMF by promoting related products using similar designs, brand names, or colour schemes, and so forth. (World Health Organization 2016). Some formula companies provide gifts to health workers or sponsor health worker training, research, and health professional association meetings to promote their products (Rollins et al. 2023) and some sales representatives from formula companies approach mothers in person (e.g., outside the Maternal and Child Health Centres) or online, and offer them CMF samples and provide advice on infant and young child feeding (Vinje et al. 2017; Hong Kong Committee of United Nations Children's Fund 2018).

Internet advertising is the most common form of CMF promotion in Hong Kong (Department of Health 2021b). Social media sites use machine learning algorithms to target consumers precisely based on their interests, engagement, and purchasing behaviour (Rollins et al. 2023). Formula companies often show product images of growing‐up milks with packaging similar to their so‐called infant and follow‐up formula (Department of Health 2017, 2021b). According to the 2019 Department of Health (DH)'s Marketing Study (2021b), brand promotion and cross‐promotion were common digital marketing strategies in Hong Kong, as most advertisements did not specifically promote formula milk products for children below 36 months after the launch of the HK Code.

People increasingly rely on influencers to make purchase decisions (Hickman et al. 2020). A study found that content created by social media users can lead to higher purchase intention than disclosed advertisements and brand posts (Mayrhofer et al. 2019). Social media allows users to react to various posts. User engagement, such as interactions (likes, comments, and shares), positively impacts on brand awareness and purchase intention (Hutter et al. 2013).

To the best of our knowledge, this is the first study that examined digital marketing strategies through social media influencers in Hong Kong and no previous study has investigated the association between digital marketing strategies applied and interactions of corresponding social media posts. As influencer marketing is a relatively new but common form of digital marketing for CMF promotion, it is important to bridge these knowledge gaps to inform breastfeeding protection policies. Therefore, this study aimed to (i) investigate the compliance of digital marketing strategies for CMF promotion through social media influencers, and formula companies' social media sites and websites with the International Code and the HK Code; (ii) identify common digital marketing strategies for formula milk promotion through these internet channels; (iii) assess the association between digital marketing strategies and readers' interactions to the social media posts; and (iv) assess cross‐promotion of formula products on internet channels.

## 2 Methods

### 2.1 Study Design

This was a content analysis of CMF digital marketing strategies of five major formula companies in Hong Kong—Mead Johnson, Friesland Campina, Nestlé, Danone, and Abbott (ranking from the highest sales volume) (Research and Markets 2019). Code violation was defined as marketing strategies involving CMF for children aged below 36 months that deviated from the International Code or the HK Code. No ethical approval was required for this study as all data were publicly available on the internet.

### 2.2 Data Collection

Materials related to CMF for children at any age were obtained from (i) CMF‐related Facebook and Instagram posts by local influencers, (ii) local formula companies' social media sites (Instagram, Facebook, and YouTube channels), and (iii) local formula companies' websites, during a period between September 23, 2021 and December 28, 2021. Digital advertisement materials over a 2‐year period from September 1, 2019 to August 31, 2021 were requested from the five formula companies through emails. All included webpages and posts were screen‐captured and saved for record and assessment, and were assigned with reference numbers for tracking. Duplicate posts with the same content on different social media platforms were identified by manual look‐up of the posts on different platforms by the same authors on the same day, and were included only once for generic descriptive statistics. The number of interactions (number of likes, comments, and shares) of each post were recorded for analysis.

An assessment form (Supporting Information S1: Appendix 1) for periodic assessment was created and based on the WHO NetCode Toolkit for monitoring the marketing of BMS (World Health Organization & United Nations Children's Fund 2017), The International Baby Food Action Network (IBFAN) Code Monitoring Kit (The International Baby Food Action Network 2019) and checklists of previous DH's CMF marketing studies (Department of Health 2017, 2021b). The form was used to assess two aspects of marketing practices covered by the Codes: (i) informational and educational (I&E) materials about infant feeding and (ii) CMF promotion to the general public and mothers (Supporting Information S1: Appendix 2: related provisions of the International Code; Supporting Information S1: Appendix 3: related provisions of the HK Code) (Health Bureau, Department of Health 2017; World Health Organization 1981).

#### 2.2.1 Social Media Posts by Influencers

Social media posts by local influencers were found by first identifying hashtags containing text strings of the names of the five companies or their brands, regardless of language (Chinese or English), position, or capitalisation, by using Facebook and Instagram's built‐in search engines. For Instagram, only hashtags with at least 100 tagged posts were included due to large number of relevant hashtags. This was not applied to Facebook, where the number of tagged posts for a particular hashtag was not available.

Second, the posts by local influencers were included if they contained the hashtags identified and that (i) were publicly available to any user, (ii) related to the five formula companies or their products, (iii) uploaded between September 1, 2019 and August 31, 2021, and (iv) from local micro‐influencers (1000–100,000 followers) or macro‐influencers (> 100,000 followers) (Kay et al. 2020), and (v) mentioned that the post's author was a parent in Hong Kong, could be identified that the post's author was a local parent through looking at his/her social media profile, tagged the Hong Kong regional social media account of the formula company, or included hashtag(s) mentioning the formula company plus adding ‘Hong Kong’ or ‘HK’ (e.g., @Friso_hk, #MeadJohnsonHK).

#### 2.2.2 Formula Companies' Social Media Sites

Formula companies' Hong Kong‐specific Facebook pages, Instagram, and YouTube channels were found by following the links on the company/brand websites or otherwise by searching the company/brand names in the built‐in search engines of the social media platforms.

Facebook and Instagram posts, and YouTube videos of the five companies uploaded between September 1, 2019 and August 31, 2021 were collected. Only videos up to 10 min in length were included.

#### 2.2.3 Formula Companies' Websites

All local websites of the five formula companies, their brands, and official online stores (if any) were observed. They were obtained by using the company/brand names or the company/brand names plus ‘online store’ as search terms in the Google search engine, with a search term ‘Hong Kong’ added to identify Hong Kong‐specific websites.

Five types of information were obtained from company/brand websites, including: (i) main page, (ii) product description pages of formula products for children below 36 months, (iii) I&E materials about infant feeding, (iv) promotional schemes, and (v) other marketing strategies such as sample provision. Only information of sales promotion type (e.g., promotional offers) was collected from the online stores as product descriptions were already obtained from the company/brand websites.

### 2.3 Data Analyses

Descriptive statistical analyses were performed on (i) compliance with the two codes, (ii) marketing strategies used, and (iii) cross‐promotion of formula products; and multivariable linear regressions were performed to examine the association between marketing strategies used in social media posts and readers' interactions of the posts.

To ensure the reliability of the material assessment, a second reviewer assessed a sample of materials. 40 materials were extracted randomly from Facebook and Instagram posts of influencers and formula companies (10 from each category) for inter‐rater reliability assessment by Cohen's Kappa. Assessments were performed on individual questions (questions 9, 15, 17, 19, 21, 22 and 23 of the assessment form in Supporting Information S1: Appendix 1) separately for identification of discrepancy in specific questions and altogether for an overall inter‐rater reliability rate.

#### 2.3.1 Compliance of Digital Marketing Strategies Through Influencers and Formula Companies With the International Code and the HK Code

The context of materials mentioned CMF for children below 36 months or brand promotion was assessed, with respect to (i) marketing practices for CMF promotion to the public and mothers, and (ii) information and education on infant feeding. The corresponding requirements according to the International Code (Supporting Information S1: Appendix 2) and the HK Code (Supporting Information S1: Appendix 3) are summarised in Supporting Information S1: Appendix 4.

#### 2.3.2 Identification of Digital Marketing Strategies

Marketing strategies found in literature (Chen et al. 2015; Han et al. 2022), NetCode (World Health Organization & United Nations Children's Fund 2017), IBFAN Monitoring Kit (The International Baby Food Action Network 2019), and DH's checklists (Department of Health 2017, 2021b) were summarised in the assessment form. Marketing strategies used in each material were assessed and coded accordingly. Frequencies and proportions of marketing strategies identified were analysed.

#### 2.3.3 Association Between Marketing Strategies Through Influencers and Formula Companies and Social Media Post Interactions

Multivariable linear regressions were conducted to examine the association between individual digital marketing strategies used in social media posts and the readers' interactions of the posts from Facebook and Instagram separately, after adjusting for the potential confounder (type of formula companies). Strategies of YouTube videos were not examined due to small sample size and zero interactions of some videos. Kruskal‐Wallis test, Chi‐Square test, and likelihood ratio test were conducted to confirm that type of formula companies was a confounder between marketing strategies and interactions of social media posts. All statistical analyses were conducted using SPSS version 26. A two‐tailed *p* < 0.05 was considered statistically significant.

#### 2.3.4 Cross‐Promotion

Cross‐promotion of formula products was examined in three steps: (i) observe the brand names, package designs, colour schemes, mascots, and slogans (if any) of formula products of different stages from the product description pages of company websites; (ii) record similarity of these among formula milk products for children above and below 36 months from the captured materials; and (iii) calculate the proportion of cross‐promotion observed. Observations of age mismatch between the child that appeared in the materials and the advertised products were also recorded and analysed.

### 2.4 Ethical Statement

No ethical approval was required for this study as all data were publicly available on the internet.

## 3 Results

### 3.1 Descriptive Statistics and Inter‐Rater Reliability

**Figure 1:**
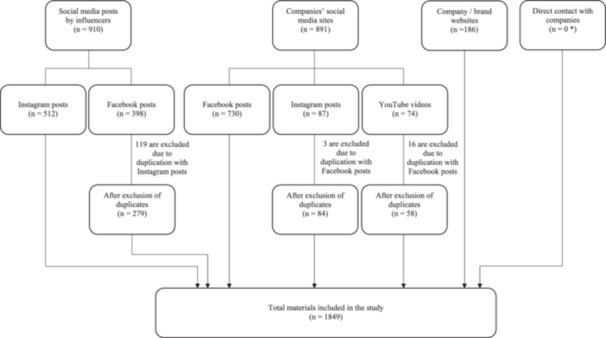
Flow chart of data collection. *No response obtained from the formula companies.

**Figure 2:**
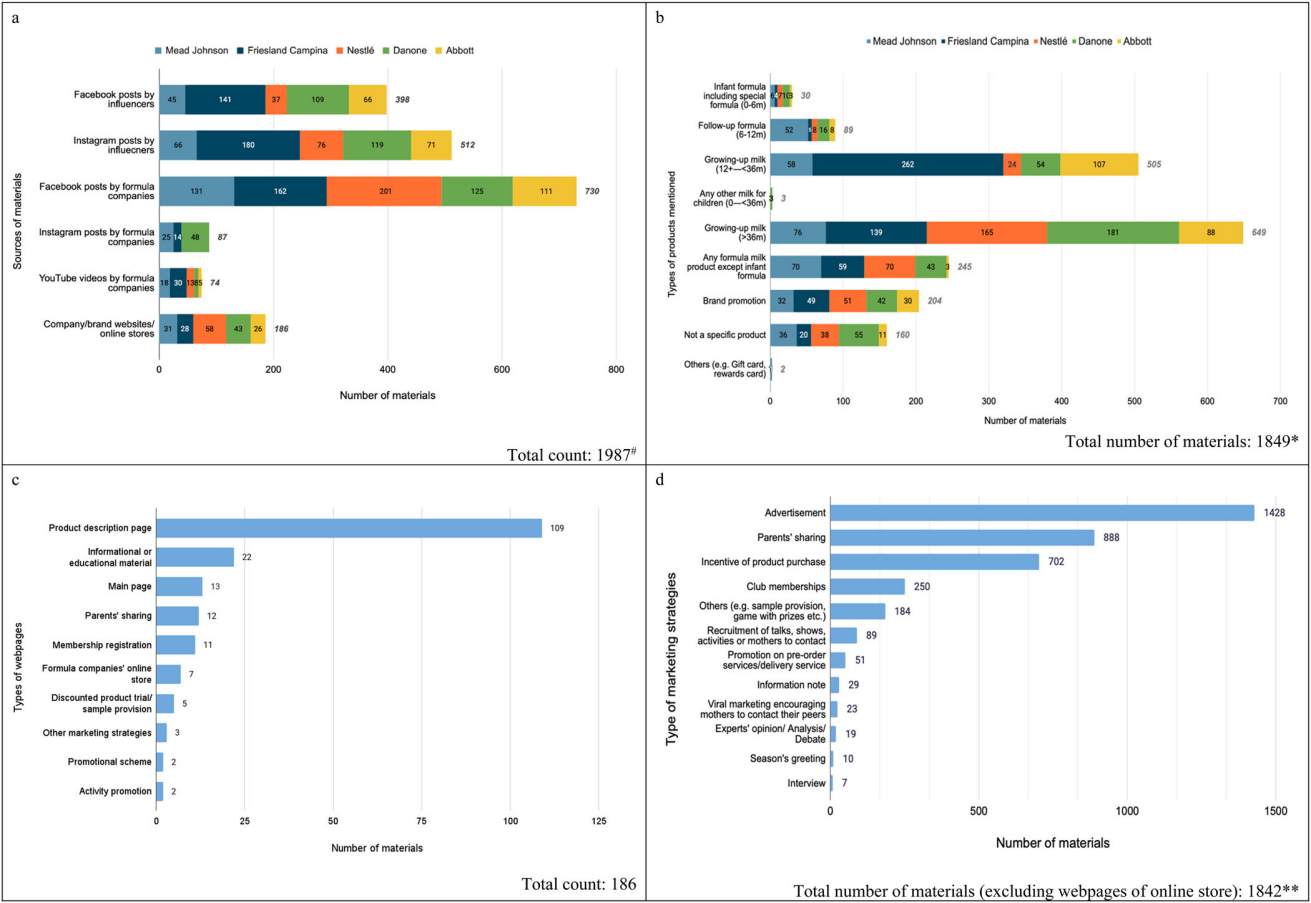
Digital marketing materials from influencers and formula companies. (a) Sources of digital marketing materials. ^#^Included duplicated materials on different channels (b) Types of products mentioned in the materials. *Some materials mentioned more than one type of product. (c) Types of webpages from formula company websites. (d) Types of marketing strategies used in the materials. **More than one type of marketing strategies were observed in some materials.

Despite reaching out to the formula companies included in this study, no digital advertisement material was obtained from them. Of 1987 materials collected, 512 (26%) and 398 (20%) materials were Instagram posts and Facebook posts by influencers, respectively; 730 (37%), 87 (4%) and 74 (4%) materials were Facebook posts, Instagram posts, and YouTube videos by formula companies, respectively; and 186 materials (9%) were webpages of company/brand websites or online stores (Figure 1). While Instagram was more commonly used by influencers for promoting CMF products than Facebook, Facebook was the most popular promotion channel for all five formula companies (Figure 2a).

After excluding duplicate materials that appeared on different social media platforms, 1849 materials of five formula companies and 37 brands (Supporting Information S1: Appendix 5) of formula milk were included in the study. 539 materials (29%) were from Friesland Campina, while 402 (22%), 363 (20%), 295 (16%) and 250 (14%) materials were from Danone, Nestlé, Mead Johnson and Abbott respectively. The most common types of products mentioned were growing‐up milk for children above 36 months (35%, 649 materials), growing‐up milk for children aged 12–36 months (27%, 505 materials), and formula milk product other than infant formula (13%, 245 materials). 183 materials (11%) were brand promotion. In total, 1036 materials (56%) mentioned products covered by the International Code (Figure 2b).

Of 186 webpages on formula company websites, the majority were product description pages (59%, 109 materials) and webpages of information and education about infant feeding (12%, 22 materials) (Figure 2c).

Regarding the promotional nature of materials, most were advertisements (78%, 1428 out of 1842 materials), parents' sharing (48%, 888 materials), and incentives of product purchase (38%, 702 materials) (Figure 2d).

The Cohen's Kappa coefficients for individual assessment items and the overall rating ranged from 0.77 to 0.93, indicating moderate to strong inter‐rater agreement (McHugh 2012).

### 3.2 Compliance of Digital Marketing Strategies Through Influencers and Formula Companies With the International Code and the HK Code

Of 1036 materials by influencers (*n* = 493) and formula companies (*n* = 543) mentioned products covered by the Codes (for children under 36 months), 924 were promotional materials (Table 1) and 112 were I&E materials (Table 2). Poor compliance to the two codes by both influencers and formula companies was observed. All influencer materials and 95% (516 of 543 materials) of company materials were found to have at least two Code violations (examples in Supporting Information S1: Appendix 6).

**Table 1:** Compliance rates of marketing practices for promoting formula milk by influencers and formula companies with the International Code of Marketing of Breast‐milk Substitutes (IC) and the voluntary Hong Kong Code of Marketing of Formula Milk and Related Products, and Food Products for Infants & Young Children (HKC).

Code items being assessed	Proportions and numbers of materials complying the two codes % (*n*)											
	Mead Johnson		Friesland Campina		Nestlé		Danone		Abbott		Overall	
	Influencers	Formula company	Influencers	Formula company	Influencers	Formula company	Influencers	Formula company	Influencers	Formula company	Influencers	Formula Company
*Denominator for #1 & #2* a	(*n* = 59)	(*n* = 104)	(*n* = 255)	(*n* = 106)	(*n* = 37)	(*n* = 91)	(*n* = 64)	(*n* = 69)	(*n* = 78)	(*n* = 61)	(*n* = 493)	(*n* = 431)
1. Not to advertise or promote products covered by the Code	0% (0)	0% (0)	0% (0)	0% (0)	0% (0)	0% (0)	0% (0)	0% (0)	0% (0)	0% (0)	0% (0)	0% (0)
2. Not to seek direct or indirect contact with expectant mothers or mothers of infants and young children (*HKC: for the purpose of promoting designated product*)	IC: 92% (54) HKC: 92% (54)	IC: 50% (52) HKC: 57% (59)	IC: 96% (244) HKC: 96% (244)	IC: 68% (72) HKC: 71% (75)	IC: 76% (28) HKC: 76% (28)	IC: 44% (40) HKC: 53% (48)	IC: 14% (9) HKC: 16% (10)	IC: 65% (45) HKC: 67% (46)	IC: 12% (9) HKC: 14% (11)	IC: 0% (0) HKC: 0% (0)	IC: 70% (344) HKC: 70% (347)	IC: 48% (209) HKC: 53% (228)
*Denominator for #3 & #4* b	(*n* = 59)	(*n* = 106)	(*n* = 255)	(*n* = 106)	(*n* = 37)	(*n* = 94)	(*n* = 64)	(*n* = 70)	(*n* = 78)	(*n* = 61)	(*n* = 493)	(*n* = 437)
3. Not to contain promotion device(s) to induce sales of formula milk	37% (22)	25% (27)	84% (215)	48% (51)	73% (27)	28% (26)	6% (4)	11% (8)	15% (12)	0% (0)	57% (280)	26% (112)
4. Not to involve provision of formula samples	98% (58)	100% (106)	100% (255)	100% (106)	100% (37)	100% (94)	98% (63)	93% (65)	53% (41)	25% (15)	92% (454)	88% (386)
**Overall compliance rate**	**0%**	**0%**	**0%**	**0%**	**0%**	**0%**	**0%**	**0%**	**0%**	**0%**	**0%**	**0%**

^a^ Denominator = Number of promotional materials mentioned products within the scope of the Code (0 – < 36 m) *excluding* webpages of online store mentioned product for 0 – < 36 m children.

^b^ Denominator = Number of promotional materials mentioned products within the scope of the Code (0 – < 36 m) *including* webpages of online store mentioned product for 0 – < 36 m children.

**Table 2:** Compliance rates of informational and educational materials on infant feeding by formula companies with the International Code of Marketing of Breast‐milk Substitutes (IC) and the voluntary Hong Kong Code of Marketing of Formula Milk and Related Products, and Food Products for Infants & Young Children (HKC).

Code items being assessed	Proportions and numbers of materials complying the two codes % (*n*)											
	Mead Johnson		Friesland Campina		Nestlé		Danone		Abbott		Overall	
	IC	HKC	IC	HKC	IC	HKC	IC	HKC	IC	HKC	IC	HKC
*Denominator for #1 & #3* a	(*n* = 18)		(*n* = 19)		(*n* = 29)		(*n* = 34)		(*n* = 12)		(*n* = 112)	
1. Contain the required information for mentioning BMS for children below 36 months old												
a. Benefits and superiority of breastfeeding	100% (18)		100% (19)		93% (27)		100% (34)		100% (12)		98% (110)	
b. Difficulty of reversing the decision not to breastfeed	0% (0)		100% (19)		59% (17)		100% (34)		100% (12)		73% (82)	
c. Maternal nutrition, and preparation for and maintenance of breastfeeding	0% (0)	—	100% (19)	—	59% (17)	—	100% (34)	—	100% (12)	—	73% (82)	—
d. Negative effect on breastfeeding of introducing partial bottle‐feeding	0% (0)	—	100% (19)	—	93% (27)	—	100% (34)	—	100% (12)	—	82% (92)	—
**Compliance rate for #1**	**0% (0)**		**100% (19)**		**59% (17)**		**100% (34)**		**100% (12)**		**73% (82)**	
*Denominator for #2* b	(*n* = 6)		(*n* = 11)		(*n* = 15)		(*n* = 12)		(*n* = 5)		(*n* = 49)	
2. Contain additional required information for mentioning infant formula												
a. Proper use of infant formula	100% (6)		100% (11)		73% (11)		100% (12)		0% (0)		82% (40)	
b. Social and financial implications of infant formula use	0% (0)		100% (11)		67% (10)		100% (12)		100% (5)		78% (38)	
c. Health hazards of inappropriate foods or feeding methods	0% (0)		100% (11)		33% (5)		100% (12)		0% (0)		57% (28)	
d. Health hazards of unnecessary of improper use of formula or BMS	0% (0)		100% (11)		73% (11)		100% (12)		0% (0)		49% (24)	
**Compliance rate for #2**	**0% (0)**		**100% (11)**		**27% (4)**		**100% (12)**		**0% (0)**		**55% (27)**	
3. Not to contain picture or text that might idealise the use of BMS	33% (6)		0% (0)		10% (3)		18% (6)		0% (0)		13% (15)	
**Overall compliance rate**	**0% (0)**		**0% (0)**		**7% (2)**		**18% (6)**		**0% (0)**		**7% (8)**	

Abbreviation: BMS, breast‐milk substitutes.

^a^ Denominator = Number of informational and educational materials by formula company mentioned products within the scope of the Code (0 – < 36 m).

^b^ Denominator = Number of informational and educational materials by formula company mentioned infant formula, brand promotion or any other milk for 0 – < 36 m children.

#### 3.2.1 Marketing Practices for Formula Milk Promotion to the General Public and Mothers

53.4% (493 materials) and 46.6% (431 materials) of the promotional materials within the scope of the Codes were from influencers and formula companies respectively. It was observed that all influencer materials and all promotional materials from formula companies had advertised or promoted CMF products for children aged less than 36 months. Besides, promotion devices such as gifts upon purchase, discounts and sample provision were commonly used to induce sales of CMF products. 43% (213 materials) of influencer materials and 74% (325 materials) of formula company promotional materials contained promotion device(s) (Table 1). Overall, the common promotion devices included gift upon purchase (40%, 215 materials), club memberships (28%, 151 materials), discounts (27%, 144 materials), and sample provision (17%, 90 materials).

Moreover, 30% (149 materials) of influencer materials and 52% (222 materials) of formula company promotional materials had sought contact with pregnant women or mothers of infants and young children (Table 1). The common purposes of seeking mothers' contact were registration for mothers' club (35%, 131 materials), offering discounts (29%, 109 materials), gifts or prises (30%, 110 materials), and product samples (25%, 92 materials). According to the HK Code, formula companies should not seek directly or indirectly personal details of children, expectant parents or parents of children under 36 months old, or invite their participation in activities including mother craft activities and baby shows, for the purpose of promoting designated products. There were only slightly less materials (146 influencer materials [30%] and 203 formula company promotional materials [47%]) violated the HK Code in this aspect.

#### 3.2.2 Information and Education on Infant Feeding

All I&E materials were from formula companies, among which 82 materials (73%) contained all the four required statements for CMF for children below 36 months old (Table 2) on the materials. The most common statement mentioned was ‘the superiority and benefits of breastfeeding’, where 98% (110 materials) of the I&E materials by formula companies mentioned this (Table 2). Of 49 I&E materials mentioning infant formula or promoting brand, only 27 materials (55%) included all four statements.

Furthermore, 97 I&E materials by formula companies (87%) mentioning products covered by the Codes contained pictures or text that might idealise the use of CMF, such as highlighting superior nutritional quality, child health benefits, and nutrients that mimic breast‐milk, and so forth (Table 2).

### 3.3 Common Marketing Strategies

**Figure 3:**
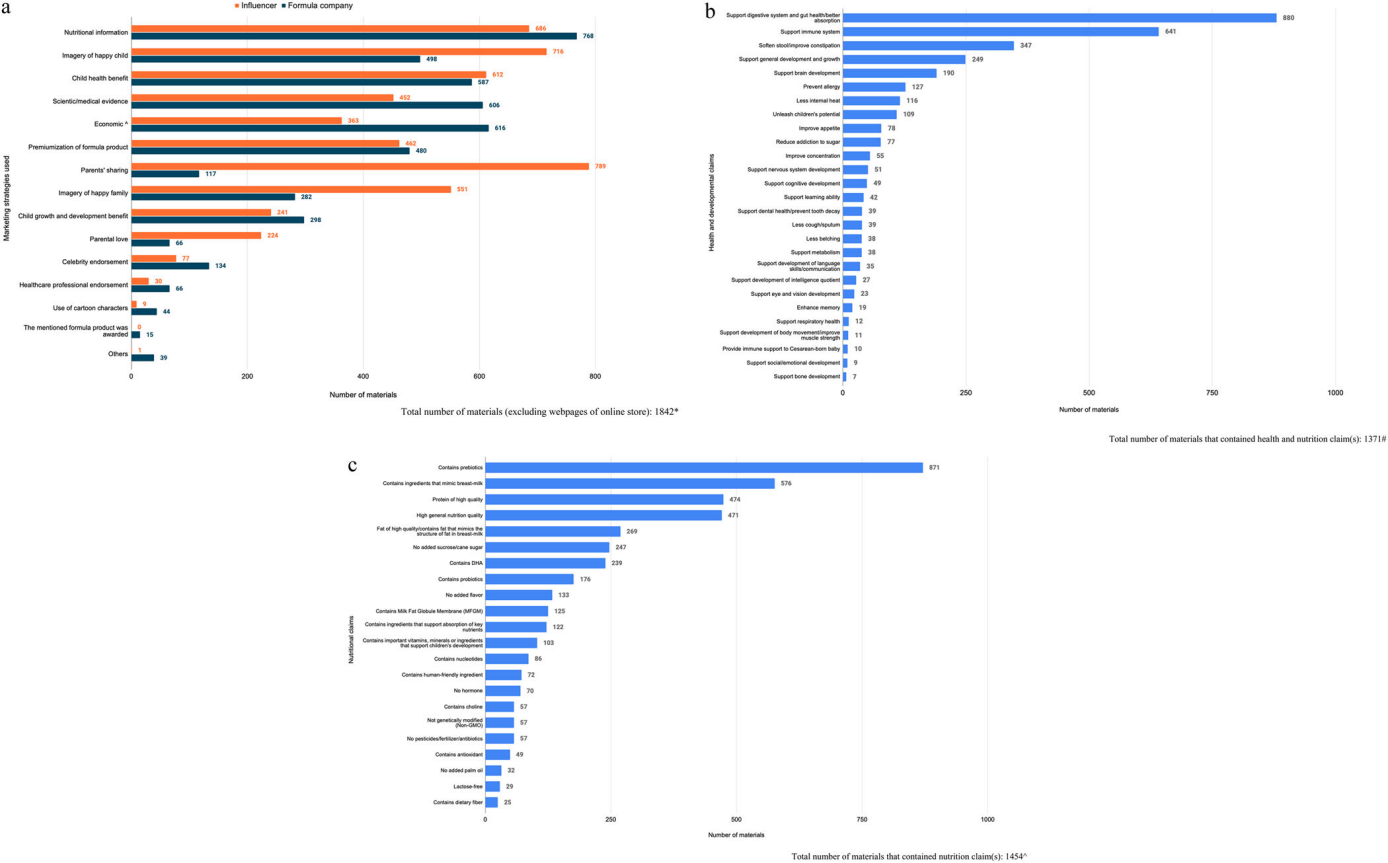
Common marketing strategies applied in the materials. (a) Types of common marketing strategies applied in the materials. *More than one type of marketing strategies were observed in some materials. ^‘Economic’ includes promotional offer, membership, new customer benefits, and mentioning value for money of the product. (b) Types of health and developmental claims mentioned in the materials. #More than one health and nutrition claims were observed in some materials. (c) Types of nutrition claims mentioned in the materials. ^More than one nutrition claims were observed in some materials.

A combination of marketing strategies (up to 11) was usually applied in one single material. Of 1842 included materials, 79% (1454 materials) highlighted nutritional information of the products (e.g., containing prebiotics, ingredients that mimic breast‐milk or high‐quality protein, etc.), 66% (1214 materials) contained imagery of a happy child, and 65% (1199 materials) highlighted benefits to child health (e.g., supporting the digestive system, gut health and immune system, etc.) (Figure 3a). Containing imagery of a happy child was the most common marketing strategy observed in company materials and parents' sharing was the most frequently observed activity in influencer materials. Details of the health and nutrition claims were presented in Figure 3b,c.

### 3.4 Association Between Marketing Strategies Through Influencers and Formula Companies and Social Media Post Interactions

A number of digital marketing strategies were found to be associated with social media post interactions (Table 3). Parents' sharing was a common and powerful strategy that was associated with more interactions in Facebook and Instagram posts by companies but not in posts by influencers because almost all influencer posts were parents' sharing. Influencers' posts that idealised the products by showing parental love were more likely to have more interactions by readers. Celebrity endorsement was associated with more interactions in all posts except Facebook posts by companies. Images of a happy family were associated with interactions in Instagram posts only which might be due to the nature of the platform that a post is accompanied with an image. However, images of a happy child were positively associated with interactions in posts by companies but not by influencers.

**Table 3:** Linear regression models for individual marketing strategies and readers' interactions with adjustment for type of formula companies.

Materials	Marketing strategies	Unstandardised coefficient	95% Confidence Interval		*p* value
Facebook posts by formula companies	Healthcare professional endorsement	−72.54	−142.45	−2.62	0.04
	Happy child	41.68	6.20	77.17	0.02
	Parents' sharing	84.10	22.13	146.07	0.01
Instagram posts by formula companies	Scientific/medical evidence	−332.96	−512.98	−152.94	< 0.001
	Celebrity endorsement	247.70	19.55	475.84	0.03
	Happy child	193.27	4.06	382.47	0.045
	Happy family	260.09	71.49	450.32	0.01
	Parents' sharing	219.41	27.99	410.83	0.03
Facebook posts by influencers	Child health and nutrition claim(s)	−128.44	−225.99	−30.89	0.01
	Premiumization	−74.86	−150.60	0.87	0.05
	Celebrity endorsement	413.51	306.22	520.79	< 0.001
	Happy child	−171.43	−275.57	−67.29	0.001
	Parental love	116.46	36.99	195.94	0.004
Instagram posts by influencers	Child health and nutrition claim(s)	−589.23	−867.73	−310.73	< 0.001
	Premiumization	−234.55	−446.09	−23.01	0.03
	Celebrity endorsement	1634.84	1333.28	1936.39	< 0.001
	Happy child	−967.72	−1448.04	−487.39	< 0.001
	Happy family	272.50	33.93	511.08	0.03
	Parental love	549.20	314.14	784.26	< 0.001

### 3.5 Cross‐Promotion

There were 183 materials (11%) indirectly promoted CMF by promoting their brands; and of 1073 materials mentioning the brands or CMF products for children over 36 months, 728 materials (68%) indirectly promoted CMF for children aged below 36 months by promoting growing‐up milk for children over 36 months of the same brand, using similar brand names, package designs or colour schemes (examples shown in Supporting Information S1: Appendix 7). Also, 8 materials (0.4%) had an age mismatch between the advertised product and the child that appeared in the material, that is, the product marketed as suitable for children aged above 36 months but the child in the material was either apparently or being described as younger than that age (examples shown in Supporting Information S1: Appendix 8).

## 4 Discussion

This study showed widespread noncompliance with the International Code and the HK Code through social media influencers and formula companies in Hong Kong, with 100% influencer materials and over 90% formula company materials having multiple Code violations, and revealed the problem of promotion in the ‘grey area’ of the Codes through influencers. Marketing strategies were applied in social media posts by influencers and formula companies to attract more readers' interactions. Similar to the CMF marketing in traditional media (Department of Health 2013, 2017, 2021b; Globalization Monitor 2019), brand promotion and cross‐promotion are common in CMF digital marketing. More than half of the materials promoted brands without specifying the age of children targeted or promoted growing‐up milk for children over 36 months of the same brand. Furthermore, some marketing practices that discouraged breastfeeding were observed in the study. For example, the advertisement often emphasised that the CMF products contained certain nutrients that mimic human milk (e.g., various types of human milk oligosaccharides and Sn‐2 palmitate, whose structure corresponds to the fatty acid in breast‐milk), or were made based on breastmilk research. Health and nutrition claims such as supporting gut health, strengthening immune system and supporting growth and development, and so forth is also a common CMF marketing strategy observed. Most of these health and nutrition claims are not scientifically substantiated and are often misleading, as many of these claims were not supported by robust scientific references or clinical trial evidence (Cheung et al. 2023). Also, formula companies often use various promotion devices to encourage the use of CMF or to promote the sales of products.

It was observed that many influencer posts during a similar time period had similar captions for promoting specific brands. Most of these posts were by micro‐influencers, who might be more influential than macro‐influencers because they usually have stronger personal connections and greater engagement with their followers. Readers might perceive posts by micro‐influencers more authentic than those by macro‐influencers and thus more prone to be influenced by micro‐influencers (Kay et al. 2020). Also, almost all influencer posts were parents' sharing which is a persuasive marketing strategy (Lee et al. 2013). Promoting CMF through influencers falls in a ‘grey area’ of both the International Code and the HK Code and should be addressed in the next revision of the Codes to protect breastfeeding.

Influencer posts and Instagram posts by formula companies involving ‘celebrity endorsement’, and influencer posts that idealised the products by showing ‘parental love’, were found to have significantly more interactions in the study. A previous study found that persuasive content of social media posts can significantly influence customer engagement (Lee et al. 2013). Product idealisation through ‘celebrity endorsement’ and ‘showing parental love’ can both be considered as a form of persuasive advertising, as they seek to influence customers through appeals to ethos or pathos (Lee et al. 2013). Another study found that customer reactions (likes and comments) to celebrity pages can be nearly double those to brand posts (Schlüschen 2016). This study also found that most celebrity influencer social media posts had more interactions than company posts. Social media are new platforms where motherhood is presented and judged; mothers' parenting practices are often being compared on social media (Mak 2015). Mothers might try to show their parental love and present themselves as good mothers who made good decisions on providing quality milk for their babies. Content related to parental love might therefore be more appealing to mothers.

Study findings on the prevalent cross‐promotion and brand promotion were consistent with several previous local studies (Department of Health 2013, 2017, 2021b; Globalization Monitor 2019). These promotional techniques were widely used by formula companies as they could effectively promote CMF products at any stage without overtly promoting products for infants and children aged below 36 months that were covered by the International Code (World Health Organization 2016). Because of the similar product packaging, mothers may not be able to readily distinguish between infant formula and growing‐up milk in the advertisements (Department of Health 2013).

This study provided an updated and more comprehensive overview of common digital marketing strategies for CMF promotion in Hong Kong and their compliance with the International Code and the HK Code, as previous DH marketing studies on CMF promotion (Department of Health 2017, 2021b) only covered marketing materials available within several months, which might have captured only snapshots of the situation within a short period, whereas this study covered a much longer period (2 years). Given the prevalence of CMF promotion through influencers, future marketing studies by the DH could consider covering the promotion by influencers, and further research could be conducted to investigate how breastfeeding practices of mothers in Hong Kong are associated with the CMF promotion through influencers.

There are several limitations in this study. First, it did not cover all digital marketing channels where CMF promotion may take place (e.g., medical‐related Facebook pages, private groups on Facebook). Second, the types of products mentioned in the materials sometimes could not be easily classified because some companies and influencers covered the stage numbers on the formula products in the media images. Third, systematic bias is of concern with content analysis being undertaken by only one reviewer. To address this concern and confirm the reliability of the material assessment, a second reviewer assessed a sample of materials.

The extensive CMF digital marketing observed occurs despite the launch of the HK Code and is possibly due to its voluntary nature and the lack of legal sanctions for noncompliance. This study has shown the extent to which the digital marketing practices of formula companies violate the two Codes; the problem of insidious yet widespread promotion of CMF through influencers; and the persisting strategies of cross‐promotion and brand promotion. These findings support the WHO's practice of adopting biennial resolutions to address changing marketing practices. There is an urgency for the Codes to be revised accordingly and the Hong Kong Government should take stronger measures, such as enshrining some provisions of the International Code and the HK Code into law with appropriate sanctions, to more effectively regulate the marketing practices of formula companies to protect breastfeeding. If the Hong Kong Government continues to fail to regulate the CMF promotion in Hong Kong, the breastfeeding rate, particularly the exclusive breastfeeding rate, may further decline. The burdens on Hong Kong's already strained healthcare system could be further increased in the long run due to suboptimal breastfeeding. It is therefore important that the Hong Kong Government takes responsibility for the proper implementation of the Codes and ensures enforcement, as outlined in the United Nations Convention on the Rights of the Child (The Office of the United Nations High Commissioner for Human Rights 1989).

## Author Contributions

W.C.N., K.H.T.Y., L.L.H. and E.A.S.N. conceptualised the study. W.C.N. and K.H.T.Y. designed the methodology of the study, validated the data. W.C.N. collected and analysed the data, conducted the investigation, wrote the original draft of the manuscript and visualised the data. K.H.T.Y. administrated and coordinated the project. E.A.S.N. acquired the funding. All authors reviewed and edited the manuscript, and have read and approved the final manuscript.

## Conflicts of Interest

The authors declare no conflicts of interest.

## Supporting information

Supporting information.

## Data Availability

All materials collected are publicly available and are available through the links attached in Supporting Information S1: Appendix 9.
