# Supplementary material for: A Content Analysis of Digital Marketing Strategies of Formula Companies and Influencers to Promote Commercial Milk Formula in Hong Kong
Source: Matern Child Nutr. 2025 Feb 25;21(3):e70007. doi: 10.1111/mcn.70007 (PMC12150114; doi:10.1111/mcn.70007)
Supplement: Supplementary file 1 — Supporting information. [file MCN-21-e70007-s001.pdf]

Appendices of "A Content Analysis of Digital Marketing Strategies of Formula Companies and Influencers to Promote Commercial Milk Formula in Hong Kong"

Authors: Wan Ching NG, Karene Hoi Ting YEUNG, Lai Ling HUI, Ka Ming CHOW, Esther Yuet Ying LAU, E. Anthony S. NELSON

DOI: 10.1111/mcn.70007

Table of Contents

Appendix 1. Formula company marketing assessment form 2

Appendix 2. Provisions of the International Code of Marketing of Breast-milk Substitute related to the study 10

Appendix 3. Provisions of the Hong Kong Code of Marketing of Formula Milk and Related Products, and Food Products for Infants & Young Children related to the study 11

Appendix 4. Assessment of compliance with the International Code of Marketing of Breast-milk Substitutes (International Code) and the voluntary Hong Kong Code of Marketing of Formula Milk and Related Products, and Food Products for Infants & Young Children (HK Code) on information and education on infant feeding and marketing practices for breast-milk promotion to the public and mothers 13

Appendix 5. List of brands of materials included in the study 14

Appendix 6. Examples of materials that had multiple Code violations 15

Appendix 7. Examples of materials that had cross-promotion 18

Appendix 8. Examples of age-mismatched between the child appeared in the material and the advertised formula milk product 21

Appendix 9. Materials included in the study (web links to a cloud drive) 22

Appendix 1. Formula company marketing assessment form

Reference number:
Date of observation (dd/mm/yyyy): __/__/____

Formula Company Marketing Assessment Form

Remarks:
* Referenced from IBFAN Code Monitoring Kit 2019 Form 5
** Referenced from WHO NetCode Toolkit Form 8
*** Referenced from checklists of Study on the Marketing of Formula Milk for Infants and Young Children in Hong Kong 2017 by DH
**** Referenced from checklists of Study on the Marketing of Formula Milk for Infants and Young Children in Hong Kong 2021 by DH
\# Investigator designed questions

SECTION I: BACKGROUND INFROMATION OF THE MATERIAL

(This section is applicable to all materials on media by formula companies or influencers that involve formula milk products for children under or above 36 months of age.)

1. Source of material: #

*If the answer is **"1"** for this question, please proceed to **Question 2**. Otherwise, please proceed to Question 3.*

1 ☐ Company/ brand/ company online store website
2 ☐ Video on YouTube by formula company
3 ☐ Post on Instagram by formula company
4 ☐ Post on Facebook by formula company
5 ☐ Post on Instagram by influencer
6 ☐ Post on Facebook by influencer
7 ☐ Media advertisement from formula company
8 ☐ Other, specify: _______

2. Type of webpage: #

1 ☐ Main page – Primary display
2 ☐ Main page – Secondary display
3 ☐ Product description page
4 ☐ Webpage of information or education related to infant feeding
5 ☐ Webpage of promotional scheme
6 ☐ Webpage of membership registration
7 ☐ Webpage of discounted product trial or sample provision
8 ☐ Webpage of activity promotion
9 ☐ Webpage of other marketing strategies, specify: __________
10 ☐ Webpage from company's online store *(skip to Q18 & Q19 and Q24 & Q25 after completing Q3 & Q4)*
11 ☐ Webpage of parents' sharing

3. (a) **Date of upload (dd/mm/yyyy): __/__/____** ☐ Not available

*(For webpage from company/brand website, if the webpage does not have date of upload, please proceed to the **Question 4**.)*

Website:
(b) Website link: ___________________
(c) Title of webpage of informational or educational material: ___________

YouTube video/ video on Facebook or Instagram:
(d) Length of video: ______ minutes ______ seconds

(cont'd Q3)
YouTube video:
(e) Number of views: _______
(f) Number of likes: _______ comments: _______

Instagram post:
(g) Number of followers of the influencer's account: ________
(h) Number of followers of formula company's account: ______
(i) Number of likes: _______ (j) comments ________

Facebook post:
(k) Number of likes or followers of the influencer's page or account: ________
(l) Number of likes of formula company's page: _______
(m) Number of likes: ________ comments: _________ shares: _________

4. To what product does the material refer? *
(a) Company:

1 ☐ Mead Johnson
2 ☐ Friesland Campina
3 ☐ Nestlé
4 ☐ Danone
5 ☐ Abbott

(b) Brand name:

1 ☐ Enfa A+
2 ☐ Enfa A+ NeuroPro
3 ☐ Enfa AII NeuroPro
4 ☐ Enfa A+ Gentle Care
5 ☐ Enfinitas
6 ☐ Nutramigen
7 ☐ Mead Johnson (All brands)
8 ☐ FRISO Gold
9 ☐ FRISO PRESTIGE
10 ☐ Organic FRISO PRESTIGE
11 ☐ FRISO (Brand promotion)
12 ☐ Nestlé NAN PRO
13 ☐ Nestlé NAN INFINIPRO
14 ☐ Nestlé NAN Organic
15 ☐ Wyeth S-26 GOLD
16 ☐ Wyeth S-26 ULTIMA
17 ☐ Wyeth PE GOLD
18 ☐ ILLUMA
19 ☐ ILLUMA LUXA
20 ☐ ILLUMA Goat
21 ☐ ILLUMA Organic
22 ☐ ILLUMA ATWO
23 ☐ ILLUMA HA
24 ☐ Cow & Gate ProAbsorb
25 ☐ Cow & Gate Happy Tummy
26 ☐ Cow & Gate Gastro-Junior
27 ☐ Aptamil Platinum
28 ☐ Aptamil Pronutra
29 ☐ Aptamil Profutura Platinum
30 ☐ Aptamil Platinum Allecure Pepti Syneo
31 ☐ Aptamil ESSENSIS HMO
32 ☐ Aptamil ESSENSIS PHP
33 ☐ Nutrilon Gold
34 ☐ Nutrilon Organic
35 ☐ Nutrilon Grass-fed Milk Source
36 ☐ Abbott Similac HMO
37 ☐ Abbott Similac Total Comfort
38 ☐ Abbott Eleva Organic
39 ☐ NONE
40 ☐ Nestlé NAN (All brands)
41 ☐ Nestlé (Wyeth) (All brands)
42 ☐ Cow and Gate (All brands)
43 ☐ Aptamil (All brands)
44 ☐ Nutrilon (All brands)
45 ☐ Abbott (All brands)
46 ☐ Abbott Similac (All brands)
47 ☐ Enfa A+ & Enfinitas
48 ☐ Enfa A+ NeuroPro & Enfinitas
49 ☐ FRISO Gold & FRISO PRESTIGE
50 ☐ FRISO PRESTIGE & Organic FRISO PRESTIGE BIO
51 ☐ ILLUMA (All brands)
52 ☐ ILLUMA & ILLUMA Organic
53 ☐ ILLUMA, ILLUMA Organic & ILLUMA ATWO
54 ☐ Wyeth S-26 GOLD & Wyeth S-26 ULTIMA
55 ☐ Cow & Gate A2 β-Casein Protein
56 ☐ Cow & Gate ProAbsorb & Cow & Gate A2 β-Casein Protein
57 ☐ Aptamil Platinum & Aptamil Pronutra
58 ☐ Aptamil Platinum & Aptamil ESSENSIS HMO
59 ☐ Aptamil ESSENSIS HMO & Aptamil ESSENSIS PHP

(c) Type of product mentioned :[* & **]

1 ☐ Infant formula including special formula (0 – 6 months)
2 ☐ Follow-up formula (6 – 12 months)
3 ☐ Growing-up milk (12+ – <36 months)
4 ☐ Any other milk for children (0 – <36 months)
5 ☐ Growing-up milk (>36 months)
6 ☐ Any formula milk product except infant formula
7 ☐ Brand promotion
8 ☐ Not a specific product
9 ☐ Follow-up formula (6 – 12 months) & Growing-up milk (12+ – <36 months)
10 ☐ Growing-up milk (12+ – <36 months) & Growing-up milk (>36 months)
11 ☐ Other, specify: _______

5. Type of promotion: [** & ****]

1 ☐ Advertisement
2 ☐ Product description
3 ☐ Club memberships
4 ☐ Incentives of product purchase
5 ☐ Promotion on pre-order service/delivery services
5a ☐ Appearance of product logo/pack shot
5b ☐ Promote formula milk product/brand
6 ☐ Expert’s opinion/ Analysis/ Debate
7 ☐ Recruitment of talks, shows, activities or mothers to contact
8 ☐ Viral marketing encouraging mothers to contact their peers about specific product or brand
9 ☐ Sweepstakes promotions
10 ☐ Parents’ sharing
11 ☐ Season’s greeting
12 ☐ Interview (e.g., with celebrities)
13 ☐ News report
14 ☐ Information note
15 ☐ Other, specify: _____________

SECTION II: CODE COMPLIANCE & MARKETING STRATEGIES AND APPREALS

(This section is applicable to all materials that involve formula milk products for infants and young children under or above 36 months. For materials that involve formula milk products for children older than 36 months or brand promotion, please also proceed to **Section III** after completing this section.)

Contents of the material:
Question 6 to 9 are for informational and education materials only. If the material is not an informational or educational materials, please skip to Question 10.

6. The Code requires certain information for informational and educational materials dealing with feeding of infants and young children. * *(For materials that involve formula milk products for children older than 36 months or not a specific product, please proceed to Question 8.*

Tick '**Missing**' if the information is not found. Tick '**Present**' if it is there.
A tick (√) under 'Missing' connotes a violation.

	Missing	Present
a. A statement on the benefits and superiority of breastfeeding	0 ☐	1 ☐
b. A statement on the negative effect on breastfeeding of introducing partial bottle feeding	0 ☐	1 ☐
c. A statement on the difficulty of reversing the decision not to breastfeed	0 ☐	1 ☐
d. A statement on maternal nutrition, and the preparation for and maintenance of breastfeeding''	0 ☐	1 ☐

(Question 7 is only applicable to the materials that involve infant formula milk products.)

7. Additional requirement for informational and educational materials mentioning infant formula. If the product is not infant formula, go to Question 8. *

Tick '**Missing**' if the information is not found. Tick '**Present**' if it is there.
A tick (√) under 'Missing' connotes a violation.

	Missing	Present
a. A statement on the proper use of infant formula	0 ☐	1 ☐
b. A statement on the social and financial implications of the use of infant formula	0 ☐	1 ☐
c. A statement on the health hazards of inappropriate foods or feeding methods	0 ☐	1 ☐
d. A statement on the health hazards of unnecessary or improper use of formula or other breastmilk substitutes	0 ☐	1 ☐

8. Does the informational and educational material contain any image, text or representation which may idealise the use of formula milk? * & ***

0 ☐ No (go to Q10)   1 ☐ Yes

9. IF YES FOR Q8, what is/are the theme(s) of the image, text or representation? (check all that apply)#
(Options are set with reference to the findings of the Study on the Marketing of Formula Milk for Infants and Young Children in Hong Kong by Department of Health in 2017, Chen et al., 2015 & Han, 2020 studies)

1 ☐ Child health benefits	2 ☐ Superior nutritional quality	3 ☐ Parent-child bonding
4 ☐ Enhance child growth/ development	5 ☐ Enhance child intelligence	6 ☐ Help building immune system
7 ☐ Human-friendly	8 ☐ Contain nutrients that mimic breast-milk	9 ☐ Bridging the gap between breastmilk and formula milk
10 ☐ Imagery of happy child	11 ☐ Imagery of happy family	12 ☐ Celebrity endorsement
13 ☐ Healthcare professional endorsement	14 ☐ That the brand is an expert in child nutrition	15 ☐ Unleash children's potential
16 ☐ Use of cartoon characters	17 ☐ Premiumization (e.g., quality assurance, foreign-made, natural, trust etc.)	18 ☐ That the brand will accompany a mother throughout the motherhood
19 ☐ That the brand will support child to grow naturally	20 ☐ Convenient	21 ☐ Other, specify______

10. Does the material contain any promotional messages or statements that implying bottle-feeding is equivalent or superior to breastfeeding? *

0 ☐ No (go to Q12)　　1 ☐ Yes

11. IF YES FOR Q10, does the item contain any term such as "materialize", "humanize" or equivalent which suggests comparison with breast-milk? ***

0 ☐ No　　1 ☐ Yes, what is the term?

	Presence
(i) "Materialize"	0 ☐ No 1 ☐ Yes
(ii) "Humanize"	0 ☐ No 1 ☐ Yes
(iii) Equivalent term, specify: _______	0 ☐ No 1 ☐ Yes

12. Does the material have any image, promotional message or statement that encourage formula milk feeding or discourage breastfeeding? ***

0 ☐ No (go to Q14)　　1 ☐ Yes

13. IF YES FOR Q12, what is/are theme(s) of the image, message, or statement? (check all that apply) #
(Options are set with reference to the findings of the Study on the Marketing of Formula Milk for Infants and Young Children in Hong Kong by Department of Health in 2017)

1 ☐ Highlights the ease of ordering and the efficiency of the delivery service	2 ☐ Highlights improvement in the composition of formula milk	3 ☐ Highlights the easy preparation of formula milk
4 ☐ Others, specify:_______		

14. Does the material contain health, growth or developmental benefit statement? ***

0 ☐ No (go to Q16)　　1 ☐ Yes

15. IF YES FOR Q14, what is/are the theme(s) of the statement? (check all that apply) #
(Options are set with reference to the findings of the Study on the Marketing of Formula Milk for Infants and Young Children in Hong Kong by Department of Health in 2017)

1 ☐ Support brain development	2 ☐ Support immune system	3 ☐ Support digestive system and gut health/ better absorption
4 ☐ Support general development and growth	5 ☐ Support eye and vision development	6 ☐ Support bone development
7 ☐ Prevent allergy	8 ☐ Support nervous system development	9 ☐ Support social/ emotional development
10 ☐ Support development of language skills/communication	11 ☐ Support development of intelligence quotient	12 ☐ Support development of body movement/ improve muscle strength or flexibility
13 ☐ Support cognitive development	14 ☐ Provide immune support to Cesarean-born baby	15 ☐ Reduce addiction to sugar
16 ☐ Enhance memory	17 ☐ Support learning ability	18 ☐ Improve concentration
19 ☐ Dental health/ prevent tooth decay	20 ☐ Less internal heat	21 ☐ Improve appetite
22 ☐ Less cough/ sputum	23 ☐ Less belching	24 ☐ Support metabolism
25 ☐ Unleash children's potential	26 ☐ The product can soften stool/ improve constipation	27 ☐ Support respiratory health
28 ☐ Other, specify:_________		

16. Does the material contain nutritional statement? ***

0 ☐ No (go to Q18) 1 ☐ Yes

17. IF YES FOR Q16, what is/are the theme(s) of the statement? (check all that apply) #
(Options are set with reference to the findings of the Study on the Marketing of Formula Milk for Infants and Young Children in Hong Kong by Department of Health in 2017)

1 ☐ High general nutrition quality	2 ☐ Protein of high quality	3 ☐ The product contains DHA
4 ☐ The product contains prebiotics (e.g., HMO, PDX, GOS, MOS, FOS, Oligofructose)	5 ☐ The product contains Milk Fat Globule Membrane (MFGM)	6 ☐ The product contains antioxidant
7 ☐ Fat of high quality/ contains fat that mimics the structure of fat in breast-milk	8 ☐ The product contains ingredients that support absorption of key nutrients	9 ☐ The product contains important vitamins, minerals or ingredients that support children's developmental needs
10 ☐ The product contains probiotics	11 ☐ The product contains choline	12 ☐ No added sucrose/cane sugar
13 ☐ No added flavor	14 ☐ The product contains human-friendly ingredient	15 ☐ No hormone
16 ☐ Not genetically modified (Non-GMO)	17 ☐ No added palm oil	18 ☐ The product contains nucleotides
19 ☐ The product contains dietary fiber	20 ☐ Lactose-free	21 ☐ No pesticides/ fertilizer/ antibiotics
22 ☐ The product contains ingredients that mimic breast-milk	23 ☐ Other, specify:______	

18. Does the item contain promotional devices to induce the sales of formula milk? ****

0 ☐ No (go to Q20) 1 ☐ Yes

19. IF YES FOR Q18, what kind of promotional device(s) is/are used? (check all that apply) #

1 ☐ Gifts upon purchase
2 ☐ Discounts
3 ☐ Cash coupon provision
4 ☐ Sample provision
5 ☐ Sweepstake promotion
6 ☐ Club membership
7 ☐ Tie-in sales
8 ☐ Free shipping
9 ☐ After-sale services
10 ☐ Promotion offer upon first purchase
11 ☐ e-coupon provision
12 ☐ Bonus points reward scheme
13 ☐Other, specify:_____

20. Does the item directly or indirectly seeking personal details of expectant parents or parents of children under the age of 36 months? ****

0 ☐ No (go to Q22) 1 ☐ Yes

21. IF YES FOR Q20, what is/are the purpose(s) of seeking the personal details? #
(Options are set with reference to the findings of the Study on the Marketing of Formula Milk for Infants and Young Children in Hong Kong by Department of Health in 2021)

1 ☐ Registration for mothers' club
2 ☐ Registration for free samples
3 ☐ Registration for discounts
4 ☐ Registration for gifts or prizes
5 ☐ Registration for hotline service
6 ☐ Registration for delivery services
7 ☐ Registration for talk/ seminar
8 ☐ Registration for competition
9 ☐ No specific purpose stated
10 ☐ Other, specify:_________

22. What kind(s) of marketing strategies and appeals does the material applied? (check all that apply) #*(Options are set with reference to Chen et al., 2015 & Han, 2020 studies)*

1 ☐ Highlights child health benefit
2 ☐ Highlights benefit to child growth and development
3 ☐ Premiumization
- 3a ☐ quality assurance
- 3b ☐ foreign-made
- 3c ☐ natural
- 3d ☐ trust

4 ☐ Economic
- 4a ☐ promotional offer
- 4b ☐ membership
- 4c ☐ new customer benefits
- 4d ☐ the product is value for money

5 ☐ Scientific/ medical evidence
- 5a ☐ breast-milk research
- 5b ☐ added nutrient constituents
- 5c ☐ research or/and innovation

6 ☐ Celebrity endorsement
7 ☐ Healthcare professional endorsement
8 ☐ Happy child
9 ☐ Happy family
10 ☐ Parental love
11 ☐ Use of cartoon characters
12 ☐ Awards
13 ☐ NONE
14 ☐ Parents' sharing
15 ☐ Others, specify:________

23. Is there any information about the recommendations on breastfeeding? For example, World Health Organization's recommendation of exclusive breastfeeding till 6 months of age, promotion of breastfeeding, or equivalent. [#]

$_0$ ☐ No $_1$ ☐ Yes

SECTION III: CROSS-PROMOTION OF FORMULA PRODUCTS

(This section is only applicable to the materials that involve promotion of formula milk products for children older than 36 months of age or brand promotion.)

24. Does the advertised formula milk product have similar color scheme, design, brand name, slogan, or mascot to the products for infants and children under 36 months of age of the same brand? [** & ****]

$_0$ ☐ No $_1$ ☐ Yes $_2$ ☐ Not applicable

25. Does the advertised formula milk product have an appearance of age mismatch between the ad and the formula milk product? [****]

$_0$ ☐ No $_1$ ☐ Yes $_2$ ☐ Not applicable

- END -

References (Assessment form)

1. The International Baby Food Action Network. (2019). *Code Monitoring Kit.* Retrieved from http://www.babymilkaction.org/wp-content/uploads/2021/04/2019-CMK-Final.pdf
2. World Health Organization & United Nation Children's Fund. (2017). *NetCode Toolkit: Monitoring the marketing of breast-milk substitutes: protocol for periodic assessments.* Retrieved from https://www.who.int/publications/i/item/9789241513494
3. Department of Health. (2017). *Media and Marketing Study on Formula Milk and Related Products for Infants and Young Children.* Retrieved from https://www.fhs.gov.hk/english/reports/files/Media_and_Marketing_Study_Report_EN.pdf
4. Department of Health. (2021). *A Study on the Marketing of Formula Milk for Infants and Young Children in Hong Kong.* Retrieved from https://www.fhs.gov.hk/english/reports/files/Ex_Sum_Marketing_Study_2021_final_full.pdf
5. Chen, Y. C., Chang, J.S., & Gong, Y.T. (2015). A Content Analysis of Infant and Toddler Food Advertisements in Taiwanese Popular Pregnancy and Early Parenting Magazines. *Journal of Human Lactation, 31*(3):458–466. https://doi.org/10.1177/0890334415576513
6. Han, S., Chen, H., Wu, Y. & Pérez-Escamilla, R. (2022). Content analysis of breast milk substititues marketing on Chinese e-commerce platforms. Maternal and Child Nutrition, 18(2):e13332. https://doi.org/10.1111/mcn.13332

Appendix 2. Provisions of the International Code of Marketing of Breast-milk Substitute related to the study

Article 4. Information and education

4.2 Informational and education material, whether written, audio, or visual, dealing with the feeding of infants and intended to reach pregnant women and mothers of infants and young children should include clear information on all the following points: (a) the benefits and superiority of breast-feeding; (b) maternal nutrition, and the preparation for and maintenance of breast-feeding; (c) the negative effect on breast-feeding of introducing partial bottle-feeding; (d) the difficulty of reversing the decision not to breast-feed; and (e) where needed the proper use of infant formula whether manufactured industrially or home-prepared. When such materials contain information about the use of infant formula, they should include the social and financial implications of its use; the health hazards of inappropriate foods or feeding methods; and, in particular, the health hazards of unnecessary or improper use of infant formula and other breast-milk substitutes. Such materials should not use any pictures or text which may idealise the use of breast-milk substitutes.

Article 5. The general public and mothers

5.1 There should be no advertising or other form of promotion to the general public of products within the scope of this Code.

5.2 Manufacturers and distributors should not provide, directly or indirectly, to pregnant women, mothers or members of their families, samples of products within the scope of this Code.

5.3 In conformity with paragraphs 1 and 2 of this Article, there should be no point-of-sale advertising, giving of sample, or any other promotion device to induce sales directly to the consumer at the retail level, such as special displays, discount coupons, premiums, special sales, loss-leaders and tie-in sales, for products within the scope of this Code. This provision should not restrict the establishment of pricing policies and practices intended to provide products at lower prices on a long-term basis.

5.4 Manufacturers and distributors should not distribute to pregnant women or mothers of infants and young children any gifts of articles or utensils which may promote the use of breast-milk substitutes or bottle-feeding.

5.5 Marketing personnel, in their business capacity, should not seek direct or indirect contact of any kind with pregnant women or with mothers of infants and young children.

Appendix 3. Provisions of the Hong Kong Code of Marketing of Formula Milk and Related Products, and Food Products for Infants & Young Children related to the study

***Bolded text were the provisions that are similar to that in the International Code**

Article 4 — Information and Education

4.2.1 A manufacturer or distributor of formula milk and formula milk related product may provide information on specific brands of formula milk and formula milk related product via electronic (e.g. websites, emails) or physical means (e.g. hotlines, at the premises of retailers or at health care facilities) upon request provided that such information –

(a) is restricted to correct and factual information;
(b) **does not include any image, text or other representation that is likely to undermine or discourage breastfeeding, that makes a comparison to breastmilk, or that suggests that the product is nearly equivalent or superior to breastmilk;**
(c) **does not promote bottle feeding;**
(d) does not convey an endorsement or anything that may be construed as an endorsement by a professional or other body, unless this has been specifically approved by the Government;
(e) be written in language appropriate to the target readership, e.g. Chinese and / or English; and
(f) **satisfies the requirements in Article 4.4.1(e)**.

4.4.1 Informational and educational materials produced or distributed by parties other than manufacturers and distributors, whether written, audio or visual, which refer to infants-and-young-children feeding and nutrition and are intended to reach the general public, expectant parents or parents of children under the age of 36 months should –

(a) contain only correct and factual information;
(b) **not include any image, text or other representation that is likely to undermine or discourage breastfeeding, that makes a comparison to breastmilk, or that suggests that the product is nearly equivalent or superior to breastmilk**;
(c) **not promote bottle feeding;**
(d) not contain the brand name, logo or trade mark of formula milk and formula milk related product nor the names of any manufacturer or distributor of formula milk and formula milk related product, except for matters concerning public health / risks (e.g. government departments may recall products where product safety is a concern, Consumer Council may publish information comparing formula milk products) or for patient care (e.g. instruction by health professionals to purchase a special formula);
(e) clearly and conspicuously explain the following matters, with reference to the age of the infants and young children and the stage of feeding in discussion and with regard to the nature of informational and educational materials made–
  (iii) where the materials are on feeding by formula milk or the use of a feeding bottle –
    (A)the benefits and superiority of breastfeeding;
    (B) the value of exclusive breastfeeding for the first 6 months followed by sustained breastfeeding up to 2 years or beyond;
    (C) why it is difficult to reverse a decision not to breastfeed;
    (D) instructions for the proper preparation and use of feeding bottle and teat, including cleaning and sterilisation of feeding utensils;
    (E) the health risks of feeding by formula milk, feeding by using a feeding bottle and teat and improper preparation of feeding bottle and teat;
    (F) explanations that powdered formula milk is not a sterile product and that to minimise the risks of serious illness, formula milk which is intended for

consumption by infants under 6 months of age should be prepared using boiled water
cooled down to no less than 70 °C;

(G) formula milk should be prepared one feed at a time and that the reconstituted formula milk should be consumed within 2 hours after preparation and any unused milk must be discarded; and

(H) the financial implication of feeding an infant with formula milk.

Article 5 — Promotion to the Public

5.1 **A manufacturer or distributor should not himself or herself, or by any other person initiated by or on his or her behalf, carry out any promotional activities involving formula milk and formula milk related products**. Such promotional activities include but are not limited to –

(a) advertising;
(b) using special displays; and
(c) **offering prizes or gifts such as samples of formula milk or formula milk related products to any person.**

5.3 **A manufacturer or distributor should not himself or herself, or by any other person on his or her behalf –**

(a) **seek directly or indirectly personal details of infants, young children, expectant parents or parents of children under the age of 36 months;**
or
(b) invite participation of infants, young children, expectant parents or parents of children under the age of 36 months in activities including baby shows, mother craft activities

for the purpose of promoting designated products

Appendix 4. Assessment of compliance with the International Code of Marketing of Breast-milk Substitutes (International Code) and the voluntary Hong Kong Code of Marketing of Formula Milk and Related Products, and Food Products for Infants & Young Children (HK Code) on information and education on infant feeding and marketing practices for breast-milk promotion to the public and mothers

<table>
<tr><th>International Code</th><th>HK Code</th></tr>
<tr><td colspan="2">Marketing practices for BMS promotion to the public and mothers</td></tr>
<tr><td colspan="2">1. Not to advertise or promote products covered by the Code</td></tr>
<tr><td colspan="2">2. Not to contain promotion device(s) to induce sales of BMS</td></tr>
<tr><td colspan="2">3. Not to involve provision of BMS samples</td></tr>
<tr><td>4. Not to seek direct or indirect contact with expectant mothers or mothers of infants and young children</td><td>4. Not to seek directly or indirectly personal details of infants, young children, expectant parents or parents of children under the age of 36 months for the purpose of promoting designated products</td></tr>
<tr><td colspan="2">Information and education on infant feeding</td></tr>
<tr><td colspan="2">1. Contain the required information for mentioning BMS for children below 36 months old</td></tr>
<tr><td colspan="2">a) Benefits and superiority of breastfeeding</td></tr>
<tr><td colspan="2">b) Difficulty of reversing the decision not to breastfeed</td></tr>
<tr><td>c) Maternal nutrition, and preparation for and maintenance of breastfeeding</td><td>-</td></tr>
<tr><td>d) Negative effect on breastfeeding of introducing partial bottle-feeding</td><td>-</td></tr>
<tr><td colspan="2">2. Contain additional required information for mentioning infant formula</td></tr>
<tr><td colspan="2">a) Proper use of infant formula</td></tr>
<tr><td colspan="2">b) Social and financial implications of infant formula use</td></tr>
<tr><td colspan="2">c) Health hazards of inappropriate foods or feeding methods</td></tr>
<tr><td colspan="2">d) Health hazards of unnecessary of improper use of formula or BMS</td></tr>
<tr><td colspan="2">3. Not to contain picture or text that might idealise the use of BMS</td></tr>
</table>

BMS, breast-milk substitutes.

Appendix 5. List of brands of materials included in the study

**Formula Companies**	**Brands**
Mead Johnson	Enfa A+<br>Enfa A+ NeuroPro<br>Enfa AII NeuroPro<br>Enfa A+ Gentle Care<br>Enfinitas<br>Nutramigen
Friesland Campina	FRISO GOLD<br>FRISO PRESTIGE<br>Organic FRISO PRESTIGE
Nestlé	Nestlé NAN PRO<br>Nestlé NAN INFINIPRO<br>Nestlé NAN Organic<br>Wyeth S-26 GOLD<br>Wyeth S-26 ULTIMA<br>Wyeth PE GOLD<br>ILLUMA<br>ILLUMA LUXA<br>ILLUMA Goat<br>ILLUMA Organic<br>ILLUMA ATWO<br>ILLUMA HA
Danone	Cow & Gate ProAbsorb<br>Cow & Gate A2 β-Casein Protein<br>Cow & Gate Happy Tummy<br>Cow & Gate Gastro-Junior<br>Aptamil Platinum<br>Aptamil Pronutra<br>Aptamil Profutura Platinum<br>Aptamil Platinum Allecure Pepti Syneo<br>Aptamil ESSENSIS HMO<br>Aptamil ESSENSIS PHP<br>Nutrilon Gold<br>Nutrilon Organic<br>Nutrilon Grass-fed Milk Source
Abbott	Abbott Similac HMO<br>Abbott Similac Total Comfort<br>Abbott Eleva Organic

Appendix 6. Examples of materials that had multiple Code violations

Example 1: An informational material from Nestlé website

Source: https://www.nestlebaby.hk/content/孩子愈肥嘟嘟，愈健康？

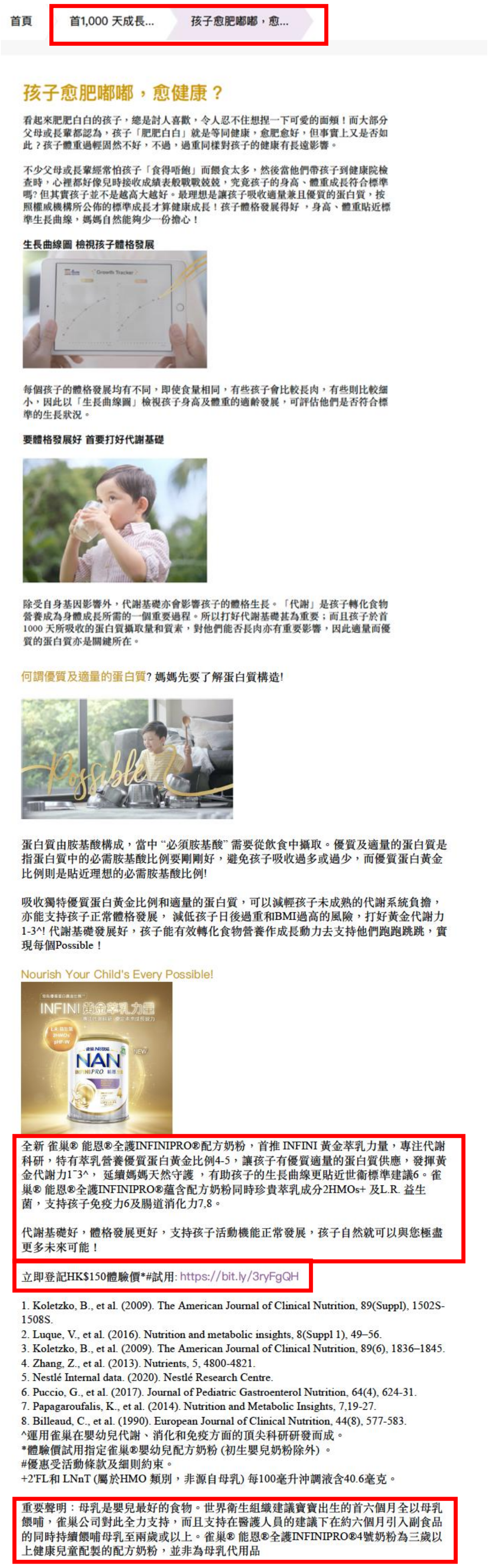

首頁 首1,000 天成長... 孩子愈肥嘟嘟，愈...

孩子愈肥嘟嘟，愈健康？

看起來肥肥白白的孩子，總是討人喜歡，令人忍不住想捏一下可愛的面頰！而大部分父母或長輩都認為，孩子「肥肥白白」就是等同健康，愈肥愈好，但事實上又是否如此？孩子體重過輕固然不好，不過，過重同樣對孩子的健康有長遠影響。

不少父母或長輩經常怕孩子「食得唔飽」而餵食太多，然後當他們帶孩子到健康院檢查時，心裡都好像兒時接收成績表般戰戰兢兢，究竟孩子的身高、體重成長符合標準嗎? 但其實孩子並不是越高大越好。最理想是讓孩子吸收適量兼且優質的蛋白質，按照權威機構所公佈的標準成長才算健康成長！孩子體格發展得好 ，身高、體重貼近標準生長曲線，媽媽自然能夠少一份擔心！

生長曲線圖 檢視孩子體格發展

每個孩子的體格發展均有不同，即使食量相同，有些孩子會比較長肉，有些則比較細小，因此以「生長曲線圖」檢視孩子身高及體重的適齡發展，可評估他們是否符合標準的生長狀況。

要體格發展好 首要打好代謝基礎

除受自身基因影響外，代謝基礎亦會影響孩子的體格生長。「代謝」是孩子轉化食物營養成為身體成長所需的一個重要過程。所以打好代謝基礎甚為重要；而且孩子於首1000 天所吸收的蛋白質攝取量和質素，對他們能否長肉亦有重要影響，因此適量而優質的蛋白質亦是關鍵所在。

何謂優質及適量的蛋白質? 媽媽先要了解蛋白質構造!

蛋白質由胺基酸構成，當中 "必須胺基酸" 需要從飲食中攝取。優質及適量的蛋白質是指蛋白質中的必需胺基酸比例要剛剛好，避免孩子吸收過多或過少，而優質蛋白黃金比例則是貼近理想的必需胺基酸比例!

吸收獨特優質蛋白黃金比例和適量的蛋白質，可以減輕孩子未成熟的代謝系統負擔，亦能支持孩子正常體格發展， 減低孩子日後過重和BMI過高的風險，打好黃金代謝力1-3^! 代謝基礎發展好，孩子能有效轉化食物營養作成長動力去支持他們跑跑跳跳，實現每個Possible！

Nourish Your Child's Every Possible!

全新 雀巢® 能恩®全護INFINIPRO®配方奶粉，首推 INFINI 黃金萃乳力量，專注代謝科研，特有萃乳營養優質蛋白黃金比例4-5，讓孩子有優質適量的蛋白質供應，發揮黃金代謝力1¯3^， 延續媽媽天然守護 ，有助孩子的生長曲線更貼近世衛標準建議6。雀巢® 能恩®全護INFINIPRO®蘊含配方奶粉同時珍貴萃乳成分2HMOs+ 及L.R. 益生菌，支持孩子免疫力6及腸道消化力7,8。

代謝基礎好，體格發展更好，支持孩子活動機能正常發展，孩子自然就可以與您極盡更多未來可能！

立即登記HK$150體驗價*#試用: https://bit.ly/3ryFgQH

1. Koletzko, B., et al. (2009). The American Journal of Clinical Nutrition, 89(Suppl), 1502S-1508S.
2. Luque, V., et al. (2016). Nutrition and metabolic insights, 8(Suppl 1), 49–56.
3. Koletzko, B., et al. (2009). The American Journal of Clinical Nutrition, 89(6), 1836–1845.
4. Zhang, Z., et al. (2013). Nutrients, 5, 4800-4821.
5. Nestlé Internal data. (2020). Nestlé Research Centre.
6. Puccio, G., et al. (2017). Journal of Pediatric Gastroenterol Nutrition, 64(4), 624-31.
7. Papagaroufalis, K., et al. (2014). Nutrition and Metabolic Insights, 7,19-27.
8. Billeaud, C., et al. (1990). European Journal of Clinical Nutrition, 44(8), 577-583.

^運用雀巢在嬰幼兒代謝、消化和免疫方面的頂尖科研研發而成。
*體驗價試用指定雀巢®嬰幼兒配方奶粉 (初生嬰兒奶粉除外) 。
#優惠受活動條款及細則約束。
+2'FL和 LNnT (屬於HMO 類別，非源自母乳) 每100毫升沖調液含40.6毫克。

重要聲明：母乳是嬰兒最好的食物。世界衛生組織建議寶寶出生的首六個月全以母乳餵哺，雀巢公司對此全力支持，而且支持在醫護人員的建議下在約六個月引入副食品的同時持續餵哺母乳至兩歲或以上。雀巢® 能恩®全護INFINIPRO®4號奶粉為三歲以上健康兒童配製的配方奶粉，並非為母乳代用品

This informational material from Nestlé website was located in a zone related to "the first 1000 days growth of children", therefore it should target parents of children aged below 36 months. The content of the material was promoting the brand "NAN INFINIPRO", yet, the picture showed a stage 4 product and the important disclaimer also mentioned stage 4 product.

It had violated six items of the International Code, including:

1. Absence of the required statements for mentioning BMS for children below 36 months of age.
2. Absence of the additional required statements for mentioning infant formula (this material was promoting the brand).
3. It highlighted the formula products of the brand had protein of good quality, HMOs and probiotics that could support children's growth, immunity and digestion, which might idealise the use of formula milk.
4. It advertised and promoted BMS product within the scope of the International Code.
5. It offered a discount for parents to purchase product sample, which is a promotion device to induce sales of formula milk.
6. It sought contact of parents of infants and young children for registering the discounted product trial.

Example 2: A Facebook post from Abbott Eleva that had 3 Code violations
Source: https://www.facebook.com/1939549412724171/posts/4193424740669949/?d=n

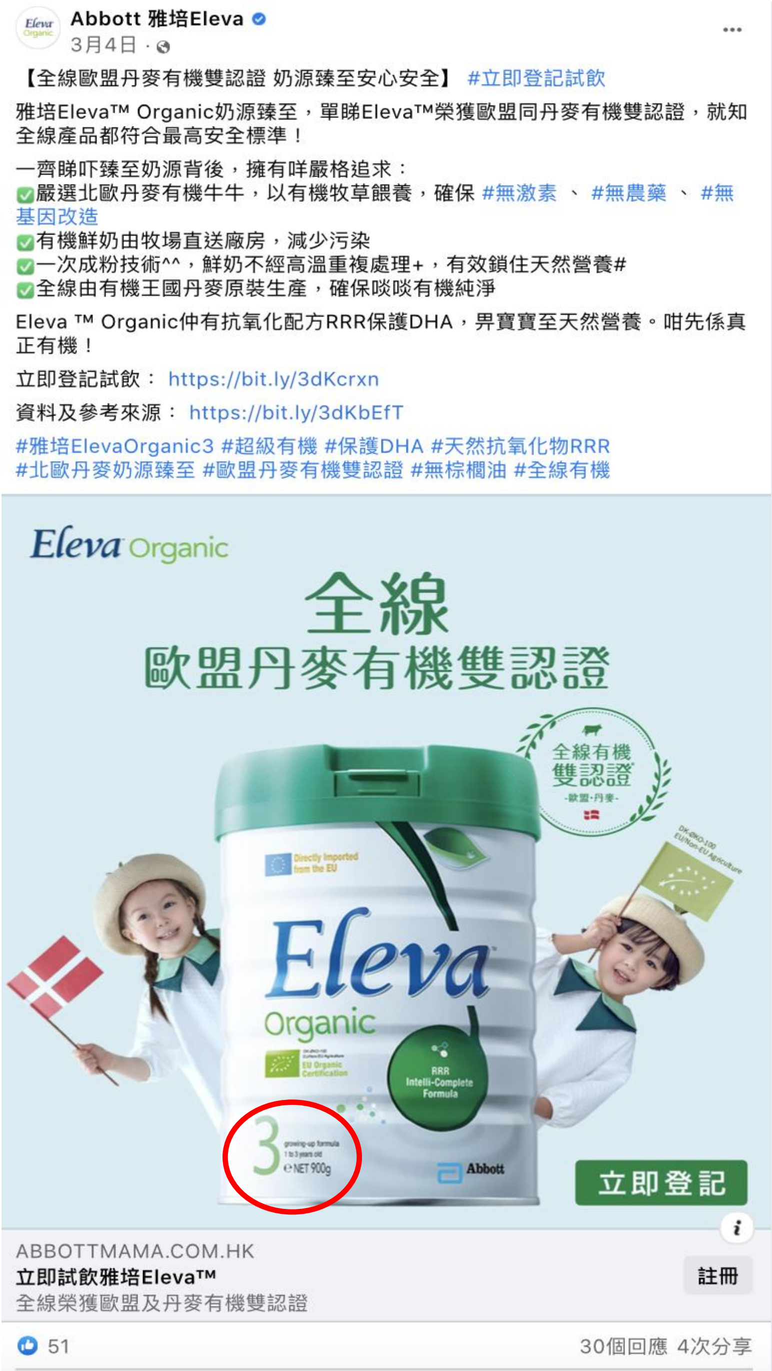

The Facebook post from Abbott had violated three items of the International Code, including:

1. Advertised and promoted BMS product within the scope of the International Code (Growing-up milk for children aged 1 to 3 years old).
2. Involved provision of product samples to mothers of young children.
3. Sought contact of mothers of young children through registration to obtain product samples.

Example 3: A local influencer's Instagram post promoting Similac from Abbott that had 3 Code violations
Source: https://www.instagram.com/p/CAjkk_jj8sz/?utm_medium=copy_link

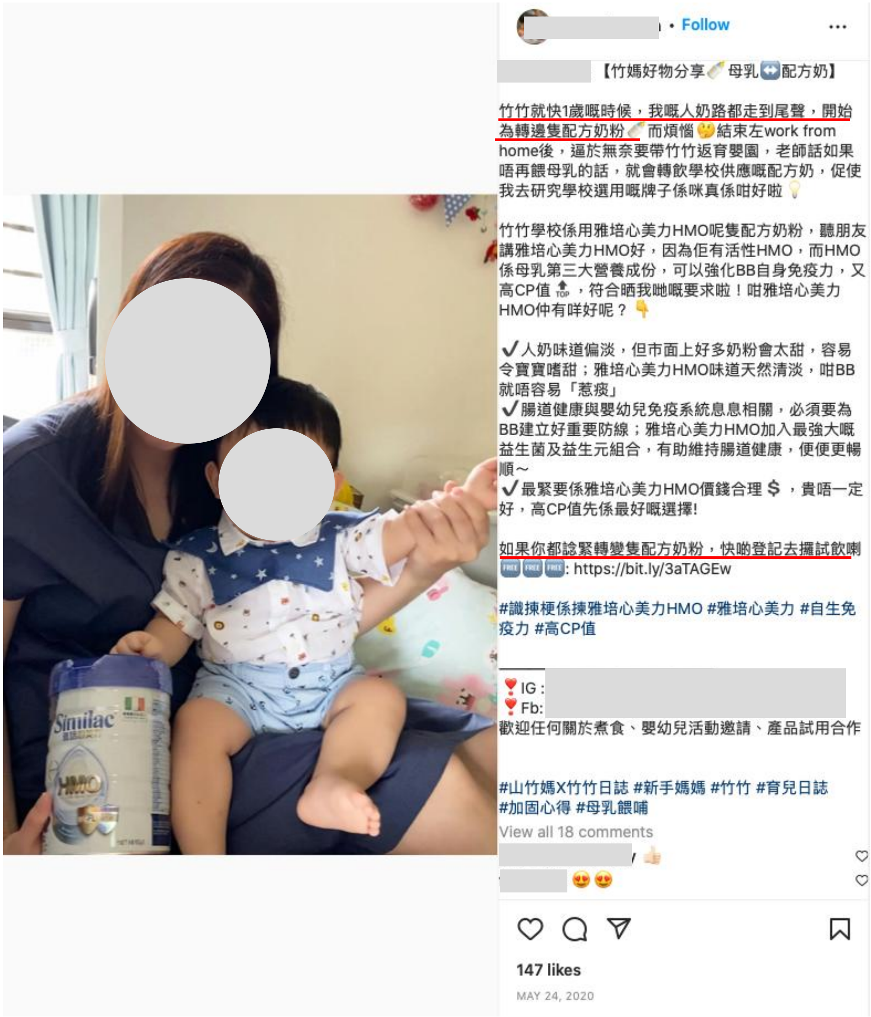

The Instagram post from a local influencer had violated three items of the International Code, including:

1. Advertised and promoted BMS product within the scope of the International Code.
2. Promoted to mothers of young children that the formula company was providing product samples for trial.
3. Helped the formula company to seek contact of mothers of young children through registering for free product samples.

Appendix 7. Examples of materials that had cross-promotion

Example 1: An influencer’s Instagram post promoting Enfinitas from Mead Johnson that had cross-promotion
Source: https://www.instagram.com/p/COMqwqkFIbG/?utm_medium=copy_link

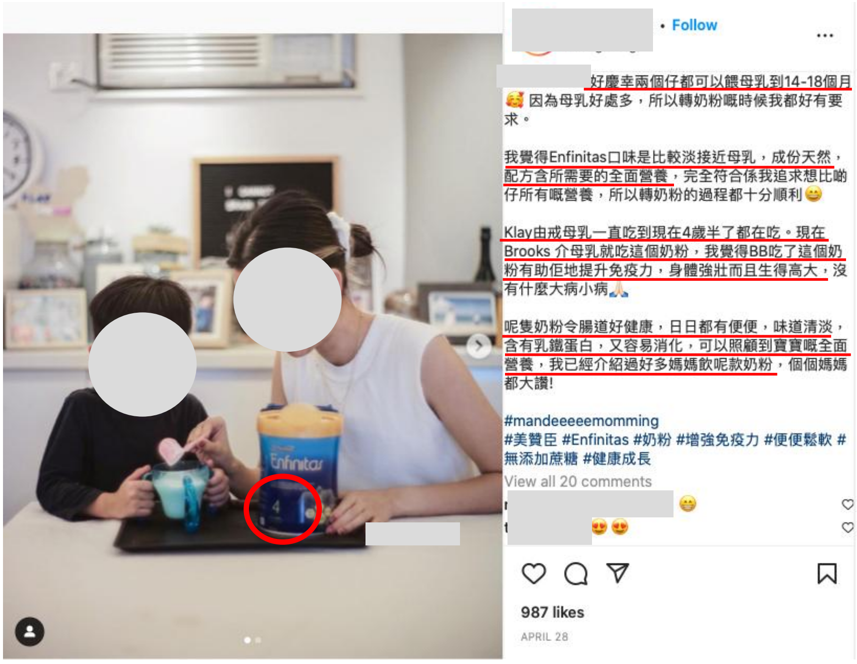

A can of growing-up milk for children above 36 months of age was shown in the photo. Yet, the caption of the post was promoting the brand instead. The influencer said that she let her children to drink the mentioned brand after they weaned breastmilk at 14 to 18 months of age. Also, the benefits to children’s health and growth, and the nutritional characteristics that she mentioned was referring to the brand instead of the growing-up milk only.

Product appearances of Enfinitas from Mead Johnson
Source: https://www.mannings.com.hk/search/?text=enfinitas

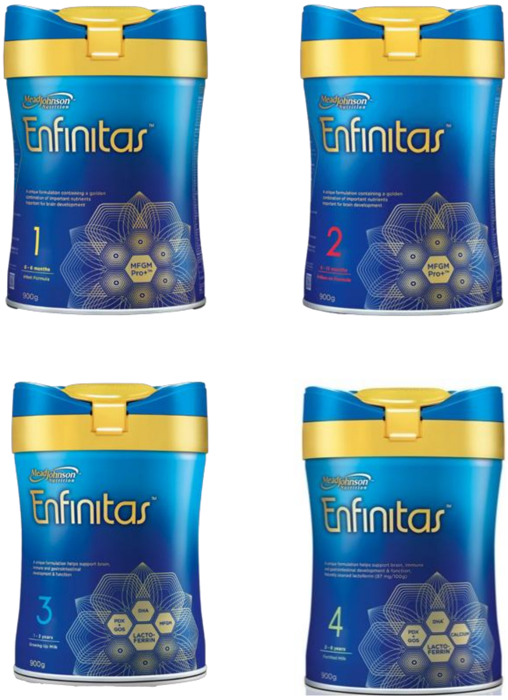

The four stages of formula milk products from Enfinitas shared the same brand name. Its growing-up milk for children above 36 months of age (Stage 4) had very similar package design to that of the BMS of the same brand (Stages 1 to 3).

Example 2: An influencer's Instagram post promoting FRISO PRESTIGE from Friesland Campina that had cross-promotion
Source: https://www.instagram.com/p/CPpKUtPDumU/?utm_medium=copy_link

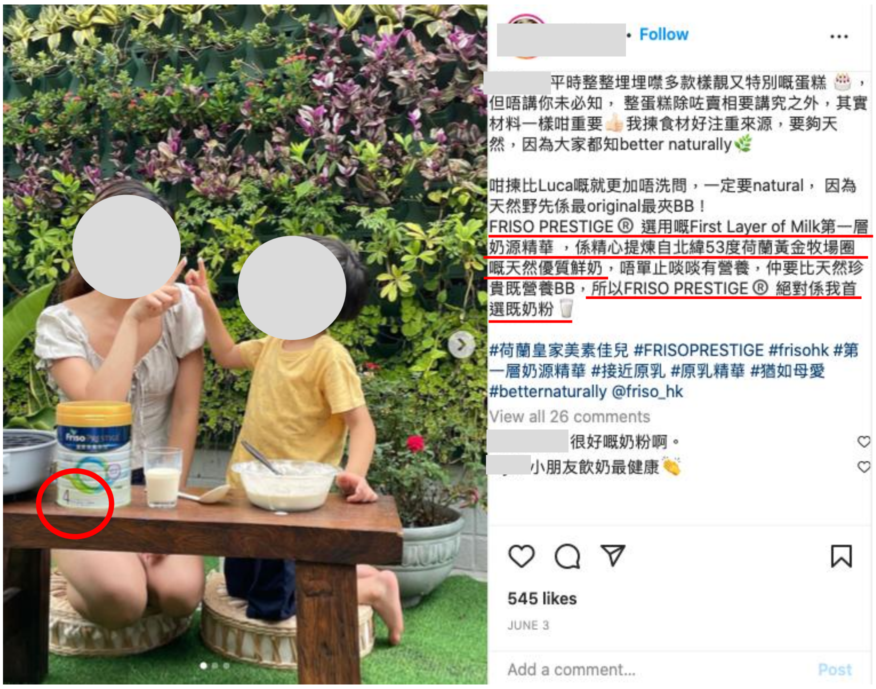

A growing-up milk for children aged above 36 months was shown in the photo. However, the post caption was not promoting the Stage 4 product only but was promoting the brand.

Product appearances of FRISO PRESTIGE from Friesland Campina
Source: https://shop.friso.com.hk

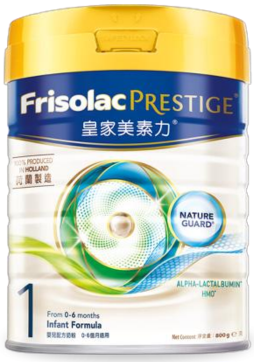

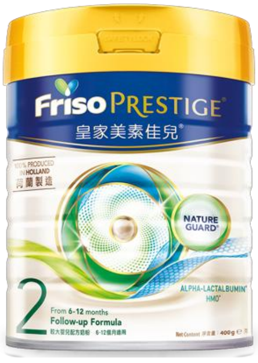

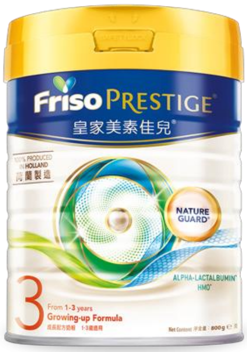

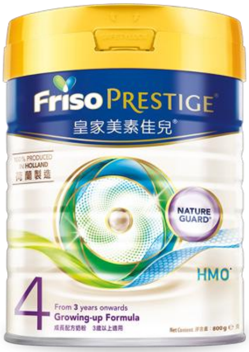

Except the infant formula (Stage 1) had a slightly different brand name, its stage 2 and 3 products shared the same brand name with the stage 4 product. All stage 1 to 4 products of FRISO PRESTIGE had very similar package designs.

Example 3: An influencer’s Facebook post promoting Cow & Gate A2 β-Casein Protein from Danone that had cross-promotion
Source: https://www.facebook.com/starfruitcayson/photos/a.125805468057539/562466744391407/

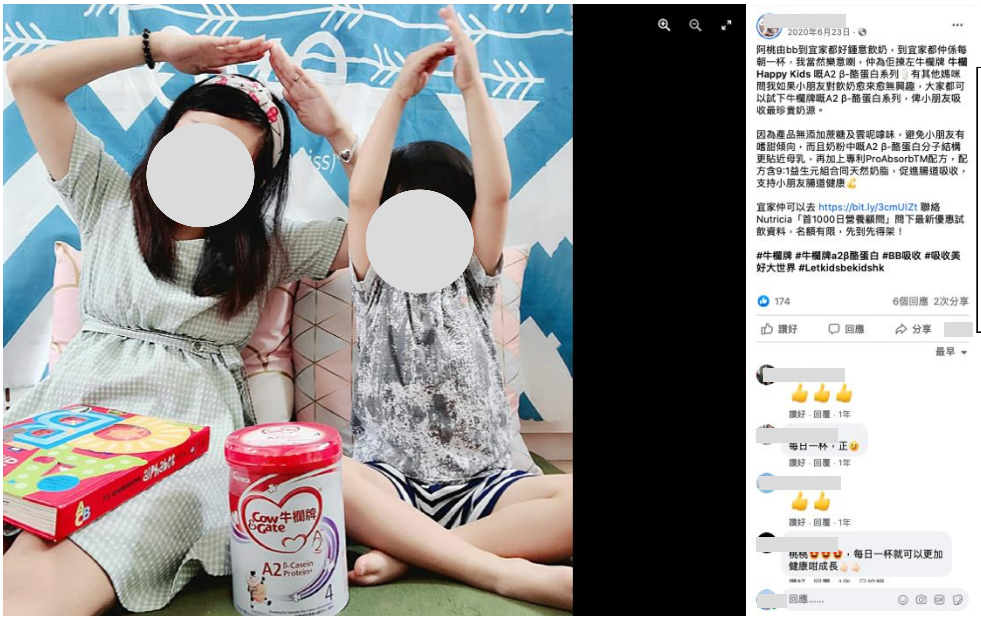

A growing-up milk product for children aged above 36 months (Stage 4) was shown in the photo. However, the caption was promoting the brand.

Product appearances of Cow & Gate A2 β-Casein Protein from Danone
Source: https://www.cghappykids.com.hk/all-products/a2-beta-casein.html

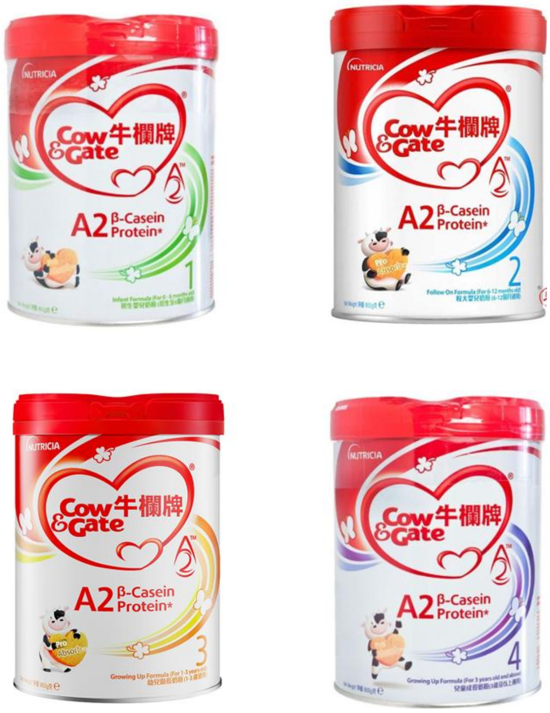

Stage 1 to 4 products of the brand shared the same brand name. They also had very similar product appearances and mascots, with only slight differences on the colours indicating different stages and the poses of the mascots.

Appendix 8. Examples of age-mismatched between the child appeared in the material and the advertised formula milk product

Example 1: A Facebook post by NeuroPro from Mead Johnson that had age-mismatched between the child appeared in the material and the advertised formula milk product
Source: https://www.instagram.com/p/CNFDf56Hy_0/

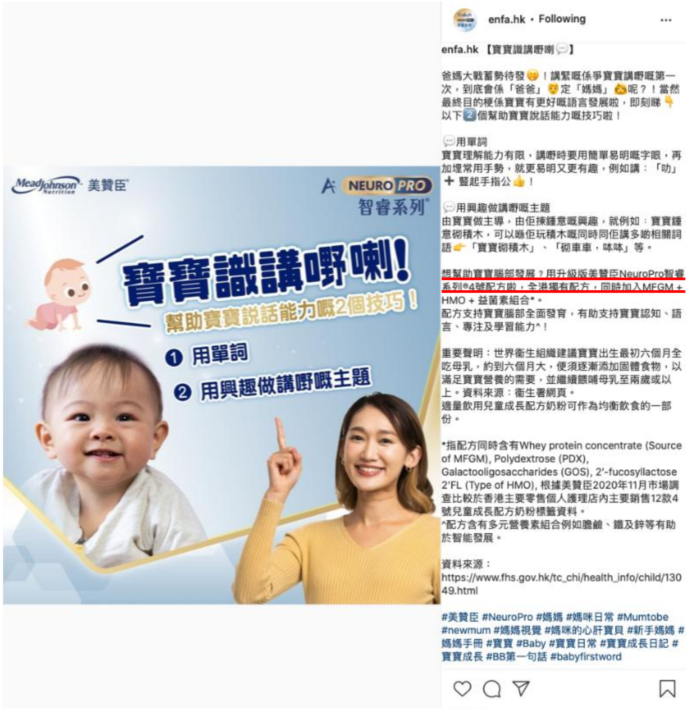

A baby that was apparently aged less than 36 months was shown in the picture. Yet, the post caption was promoting the stage 4 product of the brand, which was for children aged above 36 months.

Example 2: An influencer's Instagram post promoting ILLUMA LUXA from Nestlé that had age-mismatched between the child appeared in the material and the advertised formula milk product
Source: https://www.instagram.com/p/CQlZFDODrl_/?utm_medium=copy_link

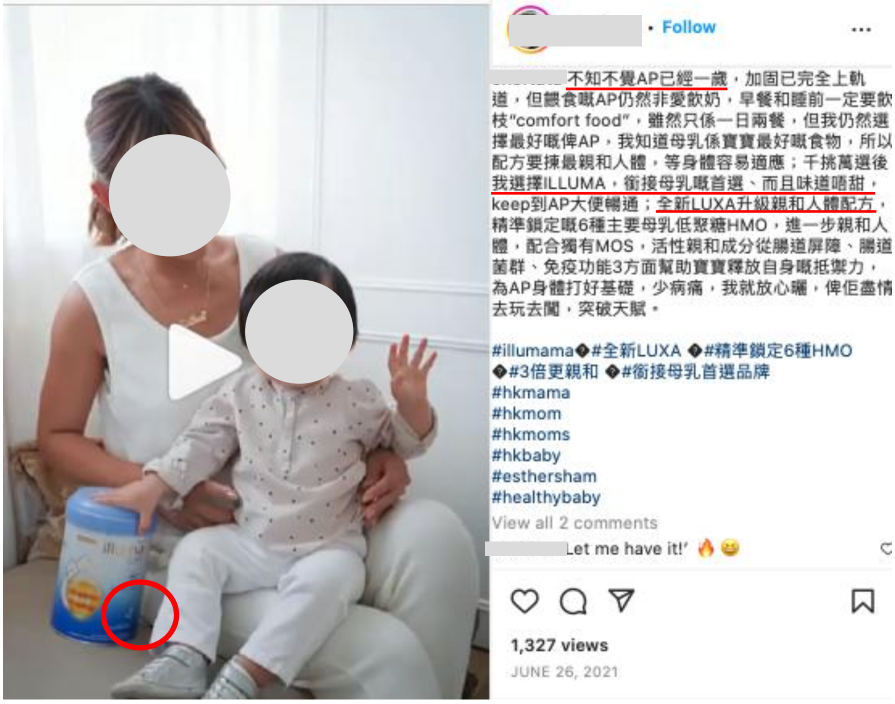

The influencer mentioned that her baby had turned one year old, but a growing-up milk for children aged above 36 months (Stage 4) was shown in the photo. Also, the post caption was promoting the brand.

Appendix 9. Materials included in the study (web links to a cloud drive)

Materials from formula company/brand websites
https://drive.google.com/drive/folders/1rl-4QC_99NSO6r1jPl1a3ln6fdotzCde?usp=sharing

Materials from formula company social media sites
https://drive.google.com/drive/folders/1zqtex-kkrOrcjE6vMwOmexYKRZfRnejs?usp=sharing

Materials from local influencers on Instagram and Facebook
https://drive.google.com/drive/folders/1c5SwRZW-zGo3j6idoljqbNpLjADF3Uh-?usp=sharing
